# Hybrid zones in the European Alps impact the phylogeography of alpine vicariant willow species (*Salix* L.)

**DOI:** 10.3389/fpls.2025.1507275

**Published:** 2025-03-20

**Authors:** Loïc Pittet, Pia Marinček, Piotr Kosiński, Natascha D. Wagner, Elvira Hörandl

**Affiliations:** ^1^ Department of Systematics, Biodiversity, and Evolution of Plants (with Herbarium), University of Göttingen, Göttingen, Germany; ^2^ Georg-August University School of Science (GAUSS), University of Göttingen, Göttingen, Germany; ^3^ Faculty of Agronomy, Horticulture and Biotechnology, University of Life Sciences, Poznań, Poland; ^4^ Institute of Dendrology, Polish Academy of Sciences, Kórnik, Poland

**Keywords:** glacial refugia, hybrid zones, phylogeography, RAD sequencing, *Salix*

## Abstract

**Introduction:**

In the European Alps, Pleistocene climate oscillations resulted in geographical range expansions and restrictions of species. Postglacial recolonizations often result in secondary contact hybridization of vicariant species, thereby creating hybrid zones with patterns of introgression. Here, we compare the genetic structure of two secondary contact hybrid zones between two vicariant willow species pairs occurring in the European Alpine System. Supplemented by morphological and ecological data, we try to understand the factors shaping the hybrid zones and their influence on geographical range filling patterns.

**Methods:**

RAD sequencing and morphometric data were used to characterize biogeographical history, genetic diversity and the hybrid zone of each species pair. Vegetation relevés and species distribution models provided ecological context and support.

**Key results:**

Results suggest that recolonization of the Alps happened from peripheral glacial refugia, resulting in broad secondary contact zones in the Eastern Alps in both species pairs. Both hybrid zones show introgression, but differ in symmetry and intensity of gene flow, in the type of introgressed loci, and in the geographical range. Habitat preferences and species distribution models do not indicate ecological barriers to recolonization.

**Conclusions:**

Hybrid zones do not only affect the genetic structure of species by gene flow and introgression, but also appear to impact the biogeographical patterns of species.

## 1 Introduction

The European Alps are the highest mountain range within the European Alpine System and harbor a great species diversity (Ozenda, 1988; Kadereit, 2017). The Alps form an arc spanning over 1200 km in the east-west direction, and about 250 km in the north-south direction (Aeschimann et al., 2004). The western part comprises the highest peaks with mountain tops above 4000 m elevation above sea level (a.s.l.) and is geologically diverse. In the eastern part the mountain chains remain below 4000 m a.s.l. and are geologically more clearly structured. The Eastern Alps are divided into the Northern and Southern Alps with calcareous bedrock, and the Central Alps with predominant siliceous bedrock, but also local calcareous substrates (**Supplementary Figure S1**). The east-west differentiation and the geological substrate influenced the different biogeographical histories and ecological niches of alpine plants (Schönswetter et al., 2005; Alvarez et al., 2009). Also, the Pleistocene climate fluctuations impacted the evolution and geographic distribution of genetic lineages (Hewitt, 2004). In Central Europe, cold-adapted plant species have survived the last glaciations in different refugial areas. These include ice-free regions within the glaciated area (Nunatak refugia hypothesis (Stehlik, 2000; Schönswetter and Schneeweiss, 2019)), unglaciated areas at the periphery of the ice sheet (peripheral refugia hypothesis (Schönswetter et al., 2005; Holderegger and Thiel-Egenter, 2009; Carnicero et al., 2022)) or lowlands (lowland refugia hypothesis (Schönswetter et al., 2005; Holderegger and Thiel-Egenter, 2009; Guerrina et al., 2022)). Additionally, other mountains of the European Alpine System that were glaciated to a lower extent or remained unglaciated played an important role as refugial areas. Further glacial refugia for cold-adapted alpine plants are usually spatially restricted and occur in the Dinarids (Spaniel and Resetnik, 2022), the Apennines, the Carpathians and the mountains of the Iberian Peninsula (Gentili et al., 2015; Schmitt, 2017). Beside these large-scale factors, the climatic gradients with increasing elevation, and microhabitat conditions in the mosaic-like subalpine and alpine vegetation influence occurrences of species.

After the last glaciation period, species have started recolonizing newly available habitats and expanding their ranges from their refugia. The current range of cold-adapted plant species often represents an incomplete transient stage (Dullinger et al., 2012b). Every species has its own recolonization rate. Location of refugia, ecological niche, dispersal ability, geographical pattern of suitable habitats, and other evolutionary processes like hybridization or polyploidization modify the recolonization rate and influence the range filling (Hewitt, 1996; Tribsch and Schönswetter, 2003; Kirchheimer et al., 2016; Smycka et al., 2022). The impact of certain factors, such as hybridization, are complex and have been largely overlooked. Hybrid zones can arise between two closely related but previously isolated species that merge their ranges during the process of recolonization in a secondary contact zone. These zones provide opportunities to assess contemporary dynamics of selection and the evolution of reproductive isolation (Barton, 2001; Petit and Excoffier, 2009). Homoploid hybrids can create adaptive combinations in the parent species by introgression and potentially promote their range expansion (Abbott et al., 2013). Adaptive introgression is likely in loci that are associated to advantageous traits, whereas loci involved in reproductive isolation are less prone to introgression (Suarez-Gonzalez et al., 2018). Testing for adaptive introgression requires not only identification of introgressed loci, but also of signatures of selection, of phenotypic changes and fitness parameters (Suarez-Gonzalez et al., 2018). Hybrid zones also facilitate gene flow between different species and introduce new genetic variation that increases the genetic diversity and may be beneficial (Rieseberg and Carney, 1998). Genomic clines will also detect intrinsic genetic incompatibilities between hybridizing species due to reproductive isolation (Gompert and Buerkle, 2012), and can therefore help us to understand the role of selection in maintaining species barriers (Fitzpatrick, 2013). However, hybrid zones can also lead to competitive interactions between the hybrids and the parent species. Hybrids may outcompete the parent species and force them to retreat to their optimal niches, resulting in ecogeographical displacement of hybrids and parents (Abbott and Brennan, 2014; Kadereit, 2015).

Here we compare the spatial genetic structure of two hybrid zones in the European Alps by investigating two sister species pairs of willows (*Salix* L.). This genus comprises approximately 33 species in the European Alps, representing a broad range of geographic patterns, habitat preferences and ploidy levels (Hörandl, 1992; Aeschimann et al., 2004; Wagner et al., 2021). Hybridization is common in *Salix* and has even been reported between species of different taxonomic sections (Argus, 1974; Hörandl et al., 2012; Gramlich et al., 2016, 2018). The two well-defined sister species pairs *S. alpina* and *S. breviserrata*, as well as *S. foetida* and *S. waldsteiniana*, are endemic to Europe (Neumann and Polatschek, 1972; Büchler, 1986; Wagner et al., 2018, 2021). They show vicariant distributions following an east-west pattern in the European Alps and adjacent mountain systems, and share a contact area situated in the Eastern Alps (Hörandl, 1992). All four species investigated are diploid (2n = 38). They are dioecious, wind- and insect pollinated and produce numerous wind-dispersed seeds (Wagner et al., 2021).

*Salix alpina* occurs in the Eastern Alps, Carpathians, and the Balkan Peninsula. *S. breviserrata* is found from the Eastern to the Southwestern Alps and in the Cantabrian Mts. Records in the Apennines (Martini and Paiero, 1988) were examined in the field (E.H.) but could not be confirmed. *Salix alpina* grows on pure limestone and dolomite, as well as base-rich silicate while *S. breviserrata* colonizes basic silicate substrates but can also occur on limestone (Merxmüller, 1952; Hörandl, 1992). Both species are ascending to erect dwarf shrubs without runners or creeping rhizomes, i.e. without clonal growth; both growing on alpine screes, rocks and in snowbeds. Morphologically, the species differ mostly by leaf margin, which is entire and ciliate for *S. alpina* but densely denticulate or serrate for *S. breviserrata*; the flowers and fruits of the two species are not differentiated (Rechinger, 1964; Martini and Paiero, 1988; Hörandl, 1992; Hörandl et al., 2012). Intermediate forms with morphological characteristics of both species have been reported in the overlapping area of the two species ranges and were interpreted to represent natural homoploid hybrids (Hörandl, 1992).

*Salix foetida* is present from the Western Alps to Tyrol as well as in the Central Apennines. Doubtful occurrence records in the Pyrenees (Blanco, 1993) were examined in the field (L.P.) but turned out to be misidentifications. *Salix waldsteiniana* occurs in the Eastern Alps, Dinarids and Balkan Peninsula; some records from the Balkan Peninsula (Rila and Vitosha Mts.) turned out to belong to *S. phylicifolia* L. s.l. (Wagner et al., 2023). *Salix foetida* primarily inhabits moist environments such as swamps, snowbeds, and rivulets, thriving on acidic silicate substrates. In contrast, *S. waldsteiniana* predominantly grows on carbonate soils but can also occur on base-rich silicates. It is less sensitive to dry conditions and is commonly found on screes, in shrubberies, and along rivulets. Both species are medium-sized, erect shrubs without clonal growth, and occur in the subalpine zone. Morphologically, *S. foetida* is characterized by dense and prominent teeth on the leaf margin that are terminated with bright glands. *Salix waldsteiniana* has sparser and less pronounced teeth with darker glands. Flowers and fruits are not differentiated, but *S. waldsteiniana* tends to form larger catkins (Rechinger, 1964; Hörandl, 1992; Hörandl et al., 2012). In the contact area of the two species ranges, transitional forms with intermediate morphological characteristics have been confirmed to be hybrids (Marinček et al., 2023).

Our study combines restriction site-associated DNA sequencing (RAD-seq) data from samples out of the whole distribution ranges with ecological niche analyses, morphological analyses and species distribution models to reconstruct the phylogeography of the four species and characterize the contact zones. The hybrid zones are analyzed to identify their genetic structure, to quantify genomic patterns of introgression and to compare the species pairs with respect to degree and symmetry of introgression. With the combined information of genetic, morphological and ecological data, we try to understand the potential factors shaping the hybrid zones and their influence on geographical range filling patterns of species.

## 2 Materials and methods

### 2.1 Sampling and molecular data generation

Leaf material of 162 individuals of *S. alpina* and *S. breviserrata* was collected from 25 locations, and leaf material of 161 individuals of *S. foetida* and *S. waldsteiniana* was collected from 33 locations, representing the whole distribution ranges of all four species, including intermediate forms. The collection and determination of pure species and intermediate forms were based on Hörandl (1992), and the authors expertise. The dataset for *S. foetida* and *S. waldsteiniana* includes 102 individuals already included in a previous study (Marinček et al., 2023). Details about locations and phenotypes are given in **Supplementary Table S1**. Leaves were dried in silica gel and voucher specimens were deposited in the Herbarium of the University of Göttingen (GOET). The DNA was extracted using the DNeasy Plant Mini Kit (Qiagen, Hilden, Germany) following a modified manufacturer’s protocol (Marinček et al., 2023). After quality check, the DNA was sent to Floragenex Inc. (Beaverton, USA) for library preparation following the protocol described in (Baird et al., 2008) and single-end RAD-sequencing. The restriction enzyme *Pst*I was used for digestion and fragments between 300 bp and 500 bp were selected.

**Figure 1:**
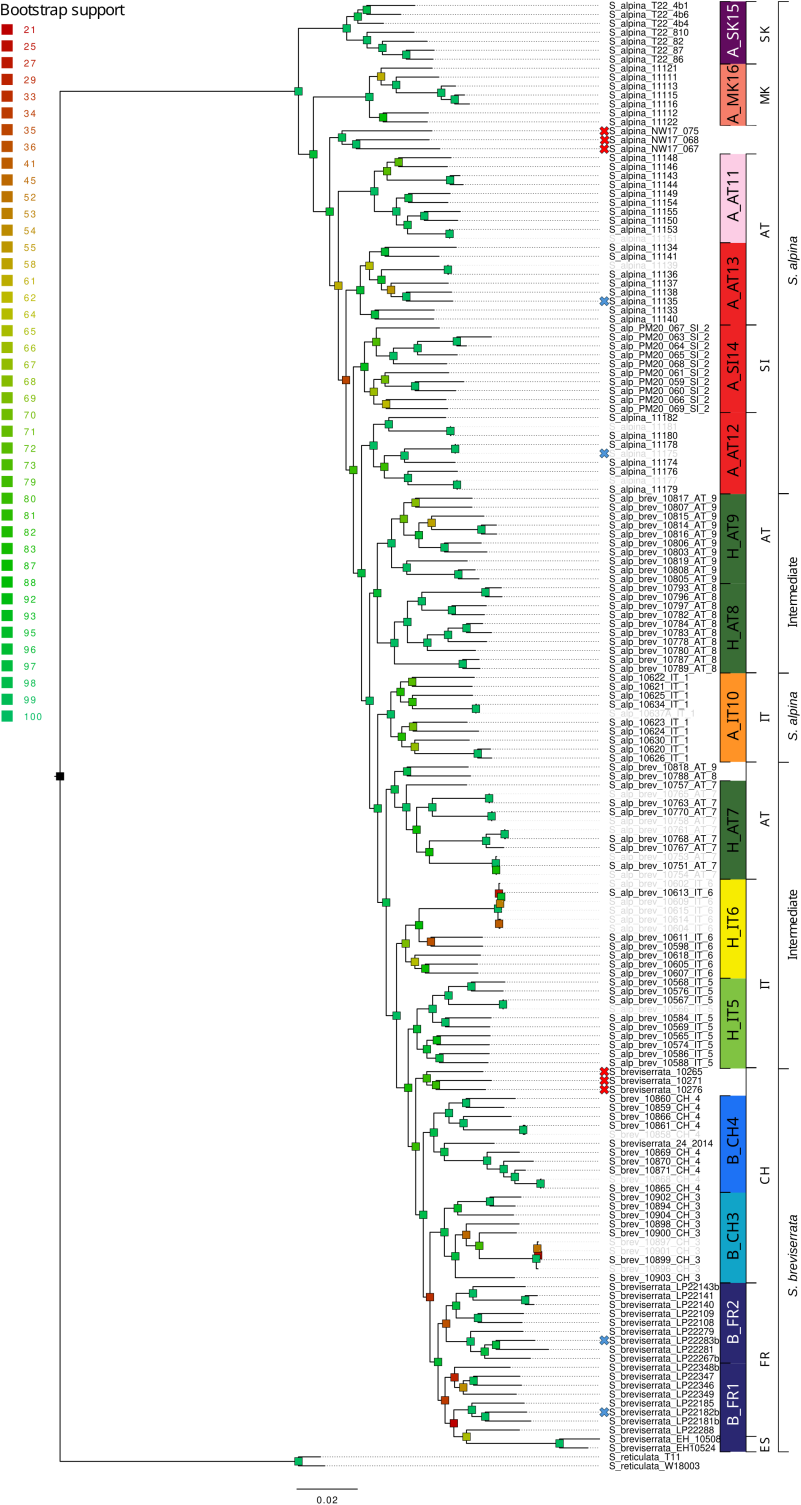
RAxML phylogeny of *Salix alpina*, *Salix breviserrata* and the intermediate forms based on 65,497 loci (576,045 concatenated SNPs). Bootstrap support is color-coded for each branch. Crosses in front of the sample name indicate that the sample was not included in the further analyses because it did not group with enough other samples (red) or because it did not pass the filtering steps (blue). Samples sharing a high proportion of identical alleles pairs (>90%) are displayed in grey. Code on the right indicates the group ID used for genetic analyses. Countries where the samples were collected and phenotypes are indicated in the last two rightmost columns, respectively (AT, Austria; CH, Switzerland; ES, Spain; FR, France; IT, Italy; MK, North Macedonia; Sl, Slovenia, SK, Slovakia).

**Figure 2:**
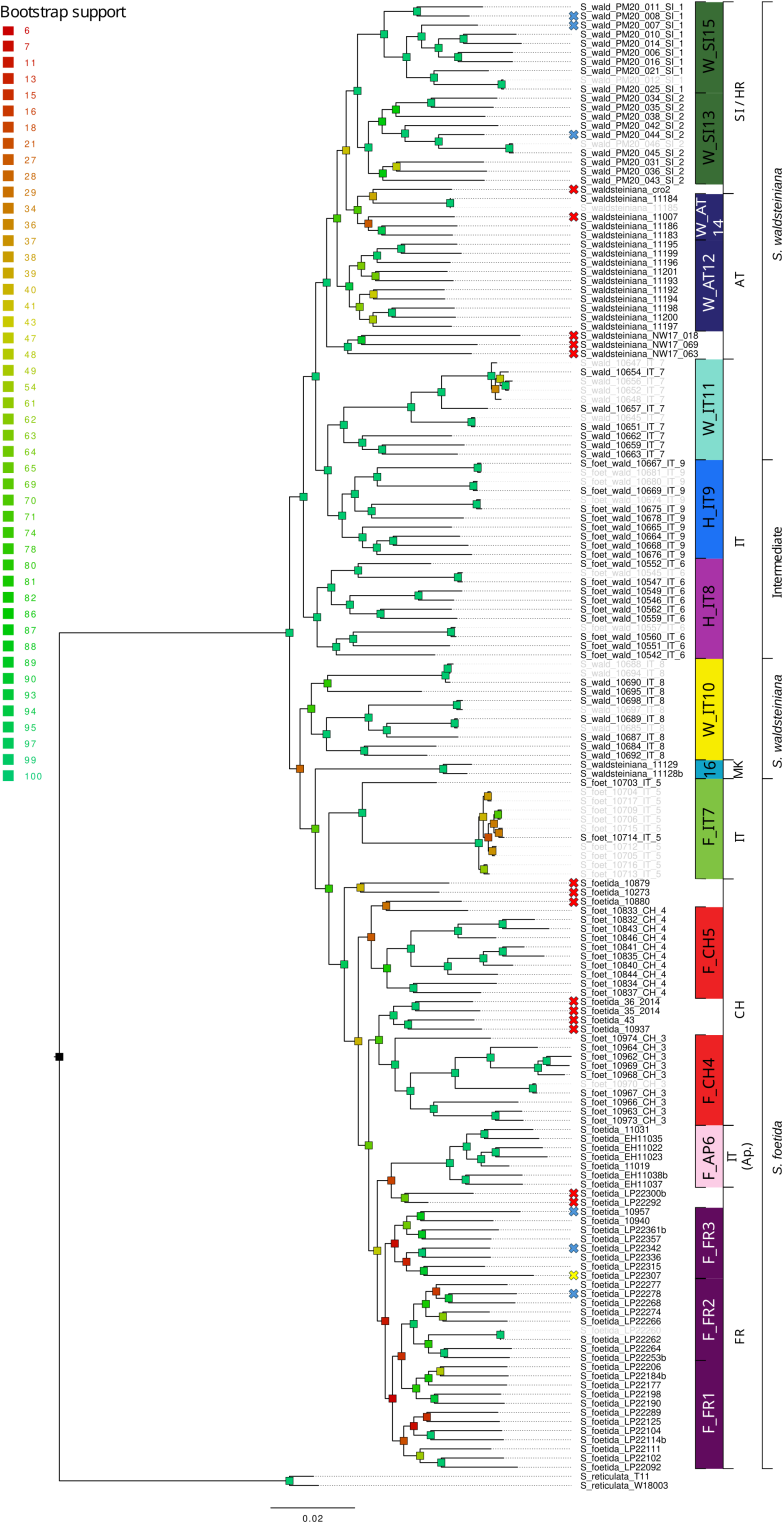
RAxML phylogeny of *Salix foetida*, *Salix waldsteiniana* and the intermediate forms based on 65,225 loci (575,194 concatenated SNPs). Bootstrap support is color-coded for each branch. Crosses in front of the sample name indicate that the sample was not included in the further analyses because it did not group with enough other samples (red), because it did not pass the filtering steps (blue), or due to potential cross-contamination (yellow). Samples sharing a high proportion of identical alleles pairs (>90%) are displayed in grey. Code on the right indicates the group ID used for genetic analyses. Countries where the samples were collected and phenotypes are indicated in the last two rightmost columns, respectively (AT, Austria; CH, Switzerland; ES, Spain; FR, France; IT, Italy; MK, North Macedonia; Sl, Slovenia, SK, Slovakia).

Each species pair was processed separately. First, a dataset including all individuals for each species pair and two accessions of *Salix reticulata* as outgroup was generated using Ipyrad v0.9.52 (Eaton and Overcast, 2020). Raw reads were demultiplexed and filtered for low-quality using default parameters and trimmed to 86 bp. Reads were clustered following the optimization as in Wagner et al. (2018), the minimum number of samples per locus was set to 50 (30%). This dataset was used to construct a phylogenetic tree that will allow us to sort out misidentifications or hybridization with other species among our samples, identify individuals with high genetic similarity and provide a phylogenetic framework for further analyses. Then, a second dataset for each species pair was generated using Stacks v2.65 (Catchen et al., 2013), which is more suitable for population genetic analysis. For this dataset, individuals were combined into groups of comparable size based on the previous phylogenetic analysis and on geographic proximity (**Figures 1**, **2**; **Supplementary Table S1**). Six individuals were removed from the *S. alpina* and *S. breviserrata* dataset because they were found in a clade with less than 6 individuals, the minimum required sample size to recover within-population genetic diversity estimates (Nazareno et al., 2017). For *S. foetida* and *S. waldsteiniana*, fourteen individuals were removed (See red crosses in **Figures 1**, **2**). A total of sixteen groups were defined for *S. alpina* and *S. breviserrata*, and sixteen groups for *S. foetida* and *S. waldsteiniana* (**Figures 1**, **2**; **Supplementary Table S1**). The *process_radtags* program with default parameters was used to demultiplex raw reads, trim the reads to 86 bp and remove low-quality reads. The quality of the resulting reads was checked using FastQC v0.11.4 (Andrews, 2010). After parameter optimization using a subset of 30 individuals and the *r80* method (Viricel et al., 2014; Paris et al., 2017; Rochette and Catchen, 2017), the *denovo_map.pl* program was used with the following parameters: -*M* 4, -*n* 4, -*m* 3 (*-M* maximum bp difference between two stacks within a sample; *-m* minimum coverage for a stack; *-n* maximum bp difference between stacks to be considered as orthologous across samples). At this point, individuals for which a large proportion of the reads represented PCR duplicates were removed. The *populations* program was further used to filter SNPs. Potential sequencing errors were removed by using a minimum minor allele count of 10 to process a SNP. Only SNPs with sequence data for at least 80% of the individuals were retained. Based on allelic balance at heterozygous sites, we checked for cross-contamination. We expected erroneous alleles coming from contamination to be much rarer than the correct allele. Individuals showing strongly unbalanced number of reads for alleles at biallelic sites were removed. Additionally, VCFtools was used to remove individuals with more than 15% missing genotypes, sites with more than 10% missing data and SNPs with excessive coverage (higher than two standard deviation above the mean) because they represented putative paralogous loci (Danecek et al., 2011). To remove linkage disequilibrium, only one SNP per locus was retained. An additional threshold for a minor allele frequency of 0.04 was applied for the dataset used with Structure, BGC, Treemix and adegenet (PCA). Further filters were applied before using Treemix and BGC to meet the analyses requirements. A summary of the pipelines, filtering steps and software used with the corresponding dataset is presented in **Supplementary Figure S2**.

### 2.2 Phylogenetic and population genetic analyses

The relationship between all individuals of a species pair was investigated using a maximum-likelihood (ML) building approach with RAxML v8.2.4 (Stamatakis, 2014). For *S. alpina* and *S. breviserrata*, the analysis was made with 64,683 RAD loci (598,650 SNPs) in a concatenated alignment of 5,648,559 bp with 17.04% missing data. For *S. foetida* and *S. waldsteiniana* 65,225 RAD loci (575,194 SNPs) in a concatenated alignment of 5,704,214 bp with 19.45% missing data were used. The GTR + Γ model of nucleotide substitution was used and a bootstrapping analysis of 100 replicates was performed. The resulting trees were visualized with Figtree v1.4.4 (Rambaut and Drummond, 2012).

Based on 35,858 unlinked and quasi-neutral SNPs in 152 individuals for *S. alpina* and *S. breviserrata*, and 36,533 unlinked and quasi-neutral SNPs in 140 individuals for *S. foetida* and *S. waldsteiniana*, a principal component analysis (PCA) was performed using the R package adegenet (Jombart, 2008). Additionally, the genetic structure among individuals was also analyzed with the Bayesian clustering approach implemented in Structure v.2.3.4 (Pritchard et al., 2000). For the analysis, the admixture model was used with correlated allele frequencies and no other prior information (Falush et al., 2003). Eight repetitions for each number of genetic clusters (K) ranging from 1 to 10 were tested. Each run had 50,000 iterations as burn-in, followed by 150,000 MCMC iterations. The R package Pophelper 2.3.1 was used to estimate the optimal number of clusters K with the Evanno method, and plot the results (Evanno et al., 2005; Francis, 2017). To infer gene flow between the different groups of a species pair, the composite-likelihood approach implemented in Treemix v.1.13 was used (Pickrell and Pritchard, 2012). Maximum Likelihood trees allowing for a predefined number of migration events were computed. The number of migration events ranged from 0 to 30 and each was tested three times. The optimal number of migration events was determined using the Evanno method. Finally, node support was estimated for the ML tree by running 100 bootstrap replicates. The R package Bite v.2 was used to find the optimal number of migration events and for bootstrap analysis (Milanesi et al., 2017). Stacks (Catchen et al., 2013) was used to compute pairwise Wright’s F_st_ between groups, and other statistics (gene diversity, nucleotide diversity, observed heterozygosity and the number of private alleles). Finally, differential patterns of introgression in hybrid groups were investigated with the Bayesian genomic cline model implemented in the software BGC (Gompert and Buerkle, 2012). It quantifies locus-specific ancestry given a hybrid index and identifies outliers based on two parameters, α and β. The hybrid index represents the proportion of an individual’s genome that is inherited from a reference population. The genomic cline center (α) gives the direction of introgression. Outliers may be involved in directional selection towards homozygotes or selection against heterozygous genotypes (Gompert and Buerkle, 2012). The cline rate (β) gives SNP frequency changes. Negative values indicate a wider cline associated with a higher introgression rate than expected. These loci may be involved in adaptive introgression. Positive values are associated with a steeper cline and reproductive isolation (Gompert and Buerkle, 2012). For the analysis, reference groups were defined based on the Q-values of Structure for K=2. Individuals with Q < 0.1 or Q > 0.9 were assigned to the respective reference groups, and the remaining were considered hybrids. The hybrid index for each admixed individual was calculated using the software BGC (Gompert and Buerkle, 2012). The genomic cline analysis was run three time independently for 600,000 MCMC iterations after a 300,000 burn-in phase. A thinning interval of 100 was used. The runs were combined, and convergence was assessed visually. Outlier loci were detected when they met both criteria, that the 95% credible intervals for α and β excluded zero and that the median of posterior distribution was outside the interval between 1-0.975/2 and 0.975/2 quantiles of the probability distribution. The R package ClineHelpR was used to visualize the output (Martin et al., 2021).

### 2.3 Morphometric analysis

Morphometric measurements were made for 154 individuals from fifteen groups of *S. alpina* and *S. breviserrata*. For *S. foetida* and *S. waldsteiniana*, 70 individuals were measured and added to the 95 individuals already measured in Marinček et al. (2023), covering fifteen groups (**Supplementary Table S2**). Up to ten entire and healthy leaves per individual were scanned with a Canon CanoScan LiDE 220 (Canon, Tokyo, Japan). Leaves were randomly selected from the herbarium specimen. Each leaf was described by six measurements and four ratios (**Supplementary Table S2**) using the software ImageJ (Schneider et al., 2012). The characteristics were chosen based on previous morphological studies on *Salix* L (Kosiński et al., 2017; Marinček et al., 2023). Leaf metrics were averaged per individual. Correlations between characteristics were checked using Spearman’s correlation coefficients, and four uncorrelated characteristics (correlation ≤ 0.7) were further standardized and analyzed by Principal Component Analysis (PCA). The area of the leaves (A), the number of teeth (T), the width-to-length ratio (BLBW) and the ratio of the total length to the length of the widest part of the leaves (BLBWP) were retained. Individuals were assigned to a category based on Structure Q-values. Individuals with less than 10% admixture (Q < 0.1 and Q > 0.9) were assigned to the respective parent species, while the other were treated as hybrids. Tests and analyses were performed with the R package MorphoTools2 (Šlenker et al., 2022).

### 2.4 Ecological niche differentiation

To examine the ecological niches of the four species outside the contact zone, we gathered vegetation relevés from the EVA Database and from our collection sites in the contact zone (**Supplementary Table S3**). Unprecise relevés, duplicates and other relevés occurring inside the contact zone were removed to avoid including wrongly determined species. A total of 1214 relevés were kept (470 for *S. alpina*, 118 for *S. breviserrata*, 160 for *S. foetida*, and 466 for *S. waldsteiniana*). Four uncorrelated Landolt indicator values (Pearson *r* ≤ 0.7) were derived from accompanying species of the target species. Landolt indicator values classify species based on their realized ecological niche using expert knowledge (Landolt et al., 2010). We used temperature (T), soil moisture variability during the growing season (VSMGS), soil pH (SpH) and soil nutrient availability (SN). The indicators unweighted mean values for each relevé were analyzed by PCA from the R package stats. Vegetation relevés from our sampling (**Supplementary Table S3**) were also plotted to compare the ecology of the different groups. Additionally, Schoener’s D index of niche overlap was calculated for each species pair using the R package Ecospat (Di Cola et al., 2017). The index ranges from 0 (no overlap) to 1 (complete overlap). For this test, background values were obtained from relevés of other mountain *Salix* species.

### 2.5 Species distribution modeling

Species occurrences were obtained from the European Vegetation Archive EVA database (Chytrý et al., 2016), Infoflora (https://www.infoflora.ch/), GBIF database (https://www.gbif.org/) and our collections. Only occurrences with precise coordinates were kept, and occurrences falling within 100m from another occurrence were discarded using the R package rangeBuilder to limit the sampling effort differences (Davis Rabosky et al., 2016). A total of 3,370 occurrences were kept (488 for *S. alpina*, 489 for *S. breviserrata*, 2,013 for *S. foetida*, and 380 for *S. waldsteiniana*). Current and historic climatic conditions were derived respectively from the CHELSA database (Karger et al., 2017; Karger et al., 2018) and PaleoView (Fordham et al., 2017). Arithmetic means of the climate variables were used to calculate bioclimatic variables. Six uncorrelated (Pearson < 0.7) bioclimatic variables were used (BIO3: Isothermality; BIO5: Mean daily maximum air temperature of the warmest month; BIO6: Mean daily minimum air temperature of the coldest month; BIO7: Annual range of air temperature; BIO14: Precipitation amount of the driest month; BIO18: Mean monthly precipitation amount of the warmest quarter). To represent the topographic variation of climate in the mountain regions, the bioclimatic variables were downscaled to a 100 m x 100 m resolution as described in (Dullinger et al., 2012a) and using a kriging with elevation as covariable. SDMs (species distribution modelling) were calibrated using the R package Biomod2 (Thuiller et al., 2023). Presence data were supplemented with four randomly selected pseudo-absence datasets. Four techniques (Generalized Linear Model, Generalized Boosting Model, Random Forest, and Generalized Additive Model) were used to model the distribution of our species. Every model was repeated four times with every pseudo-absence dataset. The calibration of the models was done with 80% of the data and, the remaining 20% were used to evaluate the models. Models with a True Skill Statistic TSS score above 0.9 were put together to generate ensemble projections of potential species distribution based on the mean of single models. Projections were made in the recent and in the Last Glaciation Maximum (LGM; 21 Kya) climatic conditions. The results of the suitable habitats were visualized in QGIS (https://www.qgis.org/).

## 3 Results

### 3.1 Phylogenetic analyses

The ML phylogenies showed well-supported topologies (**Figures 1**, **2**). The trees revealed distinct clades for all locations. For *S. alpina* and *S. breviserrata*, all locations were arranged in a nested pattern that followed a colonization from northeast to southwest. The Tatra group of *S. alpina* appeared in sister position to all the other locations (BS 100). All intermediate forms and individuals of *S. breviserrata* were derived from *S. alpina*. The clade of *S. breviserrata* was well supported (BS 100). For *S. foetida* and *S. waldsteiniana*, the individuals were divided into two well-supported clades (BS 100). Individuals from North Macedonia identified as *S. waldsteiniana* in the field were found within the clade of *S. foetida.* The clade comprising *S. waldsteiniana* individuals also comprised all phenotypically intermediate forms.

### 3.2 Population genetic analyses

**Figure 3:**
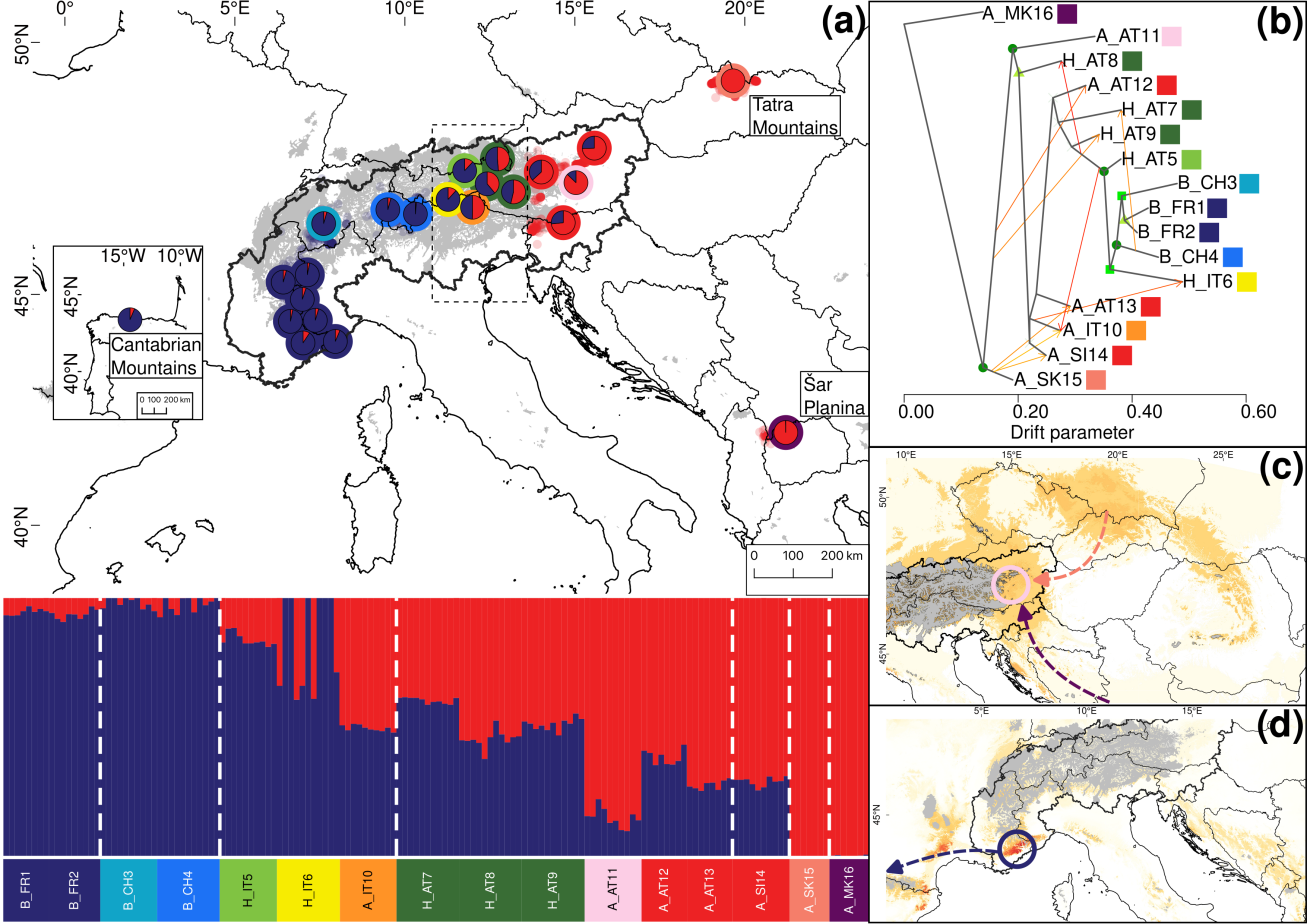
Summary of the phylogeographic analyses for 16 groups (23 locations) representing 152 individuals of *Salix alpina* and *Salix breviserrata*, based on 35,858 unlinked loci. **(A)** Map showing the results of Structure depicting genetic structure across the 23 locations for K=2. Only the average ancestry proportions of individuals for each location are displayed, providing an overview of the spatial distribution of genetic structure. Colors around the pie charts represent the group. Small dots in the background represent observations from GBIF (https://www.gbif.org, blue for *Salix breviserrata*, red for *Salix alpina*). The putative contact zone, based on phenotype only, is indicated by the dashed outline. **(B)** Gene flow between the 16 groups of *Salix alpina* and *Salix breviserrata* inferred by Treemix at the most likely number of migration m = 9. Arrows indicate the direction of the gene flow, and the color indicates the percentage of alleles that migrated from the source group (1% = yellow, 25% = orange, 50% = red). Bootstrap values for the nodes are color coded (dark green dot = 90-100%, green square = 75-90%, light green triangle = 50-75%). **(C)** Last Glaciation Maximum habitat suitability for *Salix breviserrata* and **(D)** for *Salix alpina*. Highly suitable areas are indicated in dark red. Areas covered by ice are indicated in grey. Arrows indicate potential recolonization routes. Circles indicate potential peripheral refugia. The European Commission, Eurostat (ESTAT), GISCO (2020). Countries, 2020 - Administrative Units - Dataset was used to draw the administrative boundaries **^©^** EuroGeographics.

**Figure 4:**
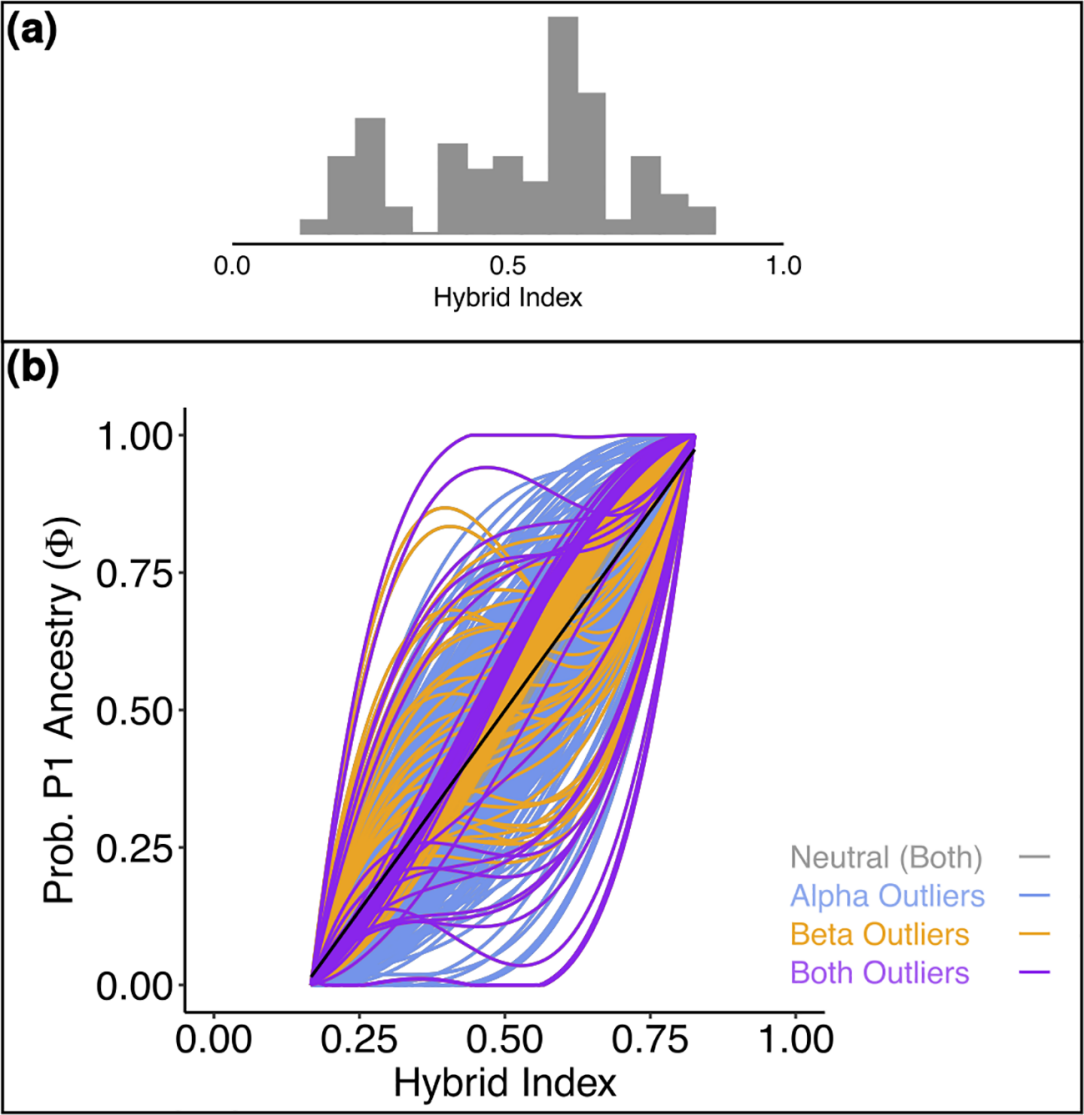
**(a)** Histogram representing the distribution of the hybrid index of the admixed individuals for *Salix alpina* and *Salix breviserrata*. Hybrid index of 0 means that the individuals is pure *Salix breviserrata*, hybrid index of 1 means that the individual is pure *Salix alpina*. **(b)** Genomic cline generated by BGC depicting the probability of P1 (*Salix alpina*) alleles given the hybrid index for 526 SNPs. Outliers are color-coded. The black line gives the null expectation based on genome-wide admixture.

For *S. alpina* and *S. breviserrata*, Structure identified K = 2 to be the optimal number of genetic clusters (**Supplementary Figure S3**). One cluster (blue) represented *S. breviserrata*, and the other cluster (red) *S. alpina* (**Figure 3A**). Groups of *S. alpina* located in the Tatra Mts. and North Macedonia showed pure ancestry, while groups in the Alps showed a moderate level of admixture with *S. breviserrata.* Intermediate forms were represented by admixture following a geographic cline. The genetic structure was confirmed with the PCA (**Supplementary Figure S5**). Treemix identified nine and 21 migration events as most likely. We chose m = 9 for clarity, under which 99.6% of the variation in the data was explained (**Supplementary Figure S7**). Overall, the topology suggested that *S. breviserrata* derived from intermediate forms, that derived themselves from *S. alpina* (**Figure 3B**). Most signals of gene flow were detected from Tatra Mts. (A_SK15) to alpine groups of *S. alpina* and intermediate forms. Three additional migration events were found between groups of the secondary contact zone. Group statistics identified most private alleles for *S. alpina* in the Tatra Mts. (A_SK15) and North Macedonia (A_MK16) (**Table 1**). The observed heterozygosity, gene diversity and nucleotide diversity were very similar for all groups, ranging from 0.185 to 0.227, from 0.177 to 0.225, and from 0.191 to 0.237, respectively. The inbreeding coefficient was slightly positive for all groups. Pairwise F_st_ values ranged between 0.046 and 0.147 (**Supplementary Table S4**). The individual hybrid index (**Figure 4a**) ranged from 0.17 to 0.82 and was strongly correlated with the longitude (Pearson’s r = 0.76, *p* < 0.001). Among the 526 loci used in BGC, 27 were negative α outliers and 51 negative β outliers (**Figure 4b**).

**Table 1:** Group statistics for *Salix alpina* and *Salix breviserrata* based on 115,347 loci.

Groups	PA	OH	GD	PI	FIS
B_FR1	0	0.18542	0.21538	0.22858	0.10801
B_FR2	0	0.20053	0.21228	0.22691	0.06536
B_CH3	13	0.20774	0.19988	0.21093	0.02055
B_CH4	1	0.20626	0.20848	0.21885	0.03573
H_IT5	0	0.21878	0.21907	0.23101	0.03386
H_IT6	36	0.2235	0.20058	0.21049	0.00026
H_AT7	15	0.21822	0.21365	0.22419	0.01327
H_AT8	6	0.21523	0.22185	0.23291	0.04837
H_AT9	1	0.21603	0.22496	0.23606	0.05507
A_IT10	1	0.21647	0.2237	0.23595	0.05193
A_AT11	38	0.20762	0.2045	0.21566	0.02141
A_AT12	0	0.22732	0.22015	0.23528	0.02032
A_AT13	0	0.21939	0.22015	0.2353	0.04024
A_SI14	0	0.21331	0.22523	0.23752	0.06418
A_SK15	91	0.185	0.17713	0.19193	0.01666
A_MK16	56	0.18497	0.17698	0.19115	0.01595

PA, number of private alleles; OH, Mean observed heterozygosity; GD, Gene diversity; PI, Nucleotide diversity; FIS, Inbreeding coefficient.

**Figure 5:**
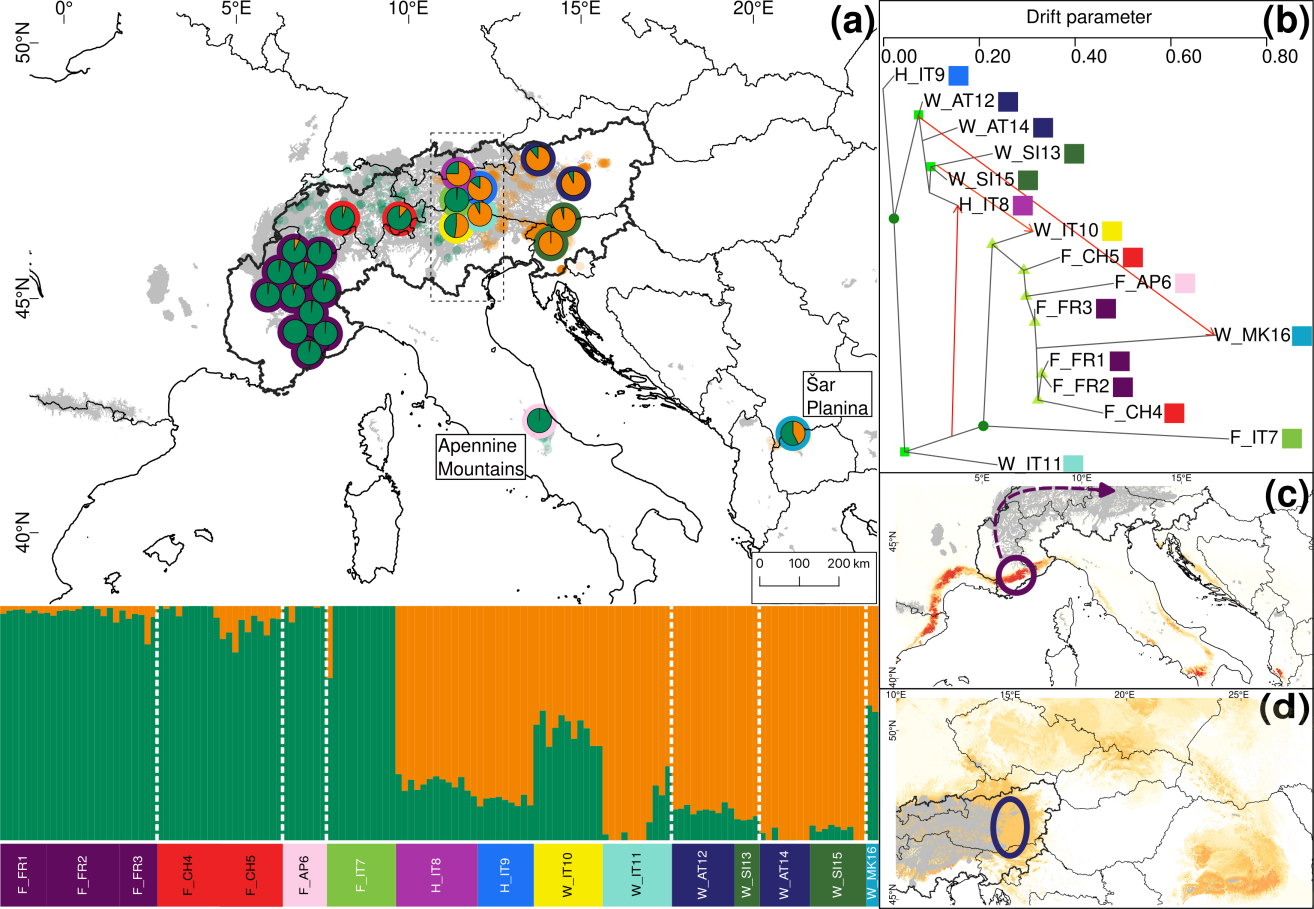
Summary of the phylogeographic analyses for 16 groups (24 locations) representing 140 individuals of *Salix foetida* and *Salix waldsteiniana*, based on 36,533 unlinked loci. **(A)** Map showing the results of Structure depicting genetic structure across the 24 locations for K=2. Only the average ancestry proportions of individuals for each location are displayed, providing an overview of the spatial distribution of genetic structure. Colors around the pie charts represent the group. Small dots in the background represent observations from GBIF (https://www.gbif.org, green for *Salix foetida*, orange for *Salix waldsteiniana*). The putative contact zone, based on phenotype only, is indicated by the dashed outline. **(B)** Gene flow between the 16 groups of *Salix foetida* and *Salix waldsteiniana* inferred by Treemix at the most likely number of migration m = 3. Arrows indicate the direction of the gene flow, and the color indicates the percentage of alleles that migrated from the source group. (1% = yellow, 25% = orange, 50% = red). Bootstrap values for the nodes are color coded (dark green dot = 90-100%, green square = 75-90%, light green triangle = 50-75%). **(C)** Last Glaciation Maximum habitat suitability for *Salix foetida* and **(D)** for *Salix waldsteiniana*. Highly suitable areas are indicated in dark red. Areas covered by ice are indicated in grey. Arrows indicate potential recolonization routes. Circles indicate potential peripheral refugia. The European Commission, Eurostat (ESTAT), GISCO (2020). Countries, 2020 - Administrative Units - Dataset was used to draw the administrative boundaries **^©^** EuroGeographics.

**Figure 6:**
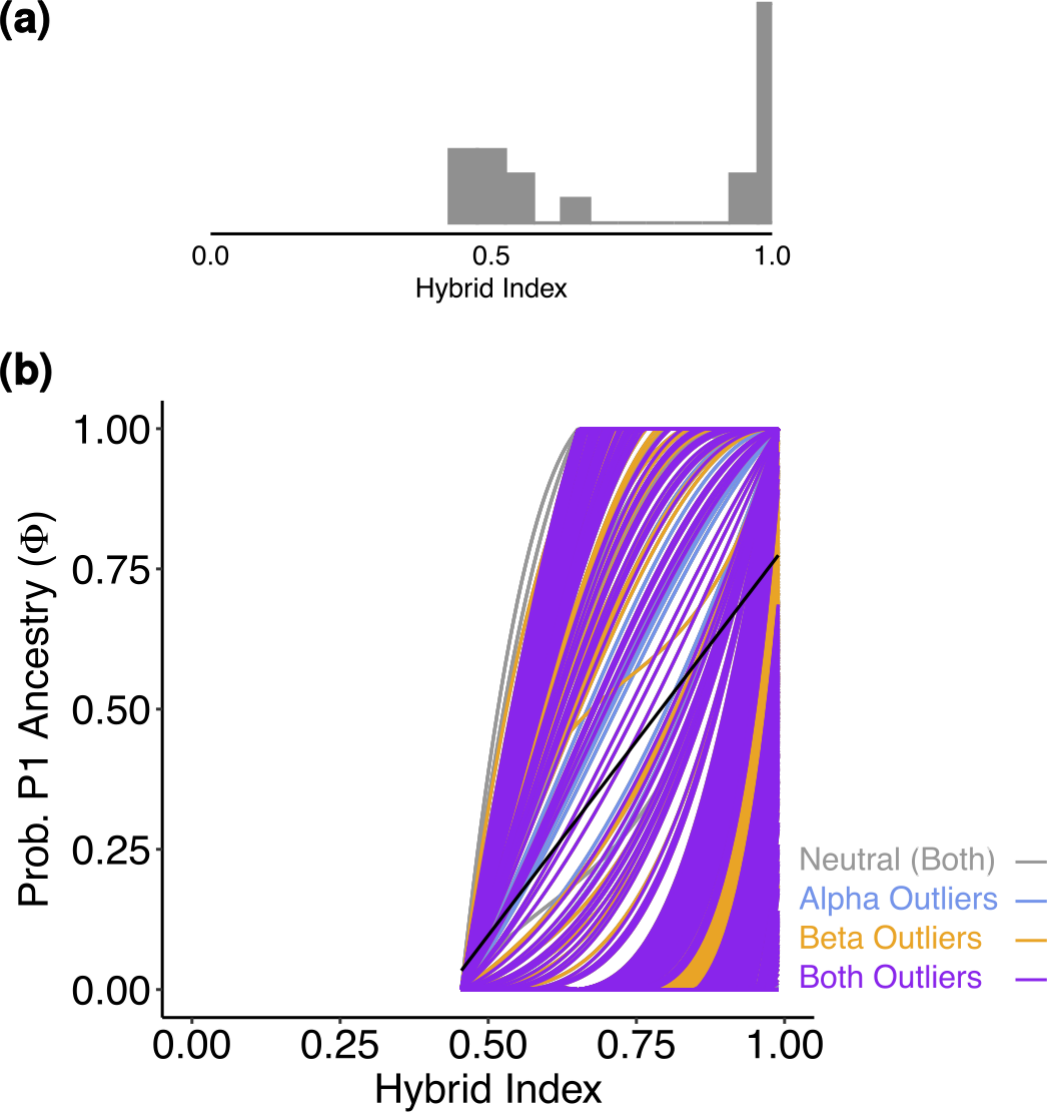
**(A)** Histogram representing the distribution of the hybrid index of the admixed individuals for *Salix foetida* and *Salix waldsteiniana*. Hybrid index of 0 means that the individuals is pure *Salix foetida*, hybrid index of 1 means that the individual is pure *Salix waldsteiniana*. **(B)** Genomic cline generated by BGC depicting the probability of P1 (*Salix waldsteiniana*) alleles given the hybrid index for 1167 SNPs. Outliers are color-coded. The black line gives the null expectation based on genome-wide admixture.

For *S. foetida* and *S. waldsteiniana*, K = 2 was identified to be optimal by Structure (**Supplementary Figure S4**). One cluster (green) was assigned to *S. foetida*, for which individuals outside the contact zone showed no or low level of admixture. The other cluster (orange) represented *S. waldsteiniana* and was pure only in Slovenia (**Figure 5A**). Individuals identified as *S. waldsteiniana* that were sampled in Italy, Austria, and North Macedonia, as well as the intermediate forms showed some level admixture. The main genetic clustering was confirmed by the PCA (**Supplementary Figure S6**). In the Treemix analysis, three migration events were detected to be optimal, and the resulting tree explained 99.5% of the total variation (**Supplementary Figure S8**). The overall topology suggested an early divergence between *S. foetida* and *S. waldsteiniana* (**Figure 5B**). Migration edges were found from an ancestral population of *S. waldsteiniana* to North Macedonia (W_MK16), from Slovenian *S. waldsteiniana* (W_SI13) to *S. waldsteiniana* in the secondary contact zone (W_IT10), and from the ancestry group of *S. foetida* to intermediate forms (H_IT8). Stacks found 650 private alleles for *S. foetida* in the secondary contact zone (F_IT7); other groups ranged from 78 to 0. The observed heterozygosity, gene diversity and nucleotide diversity were moderate, ranging from 0.162 to 0.222, from 0.113 to 0.215 and from 0.148 to 0.227, respectively (**Table 2**). Finally, the inbreeding coefficient was slightly negative for two groups and slightly positive for the rest. Pairwise F_st_ values ranged between 0.037 and 0.231 (**Supplementary Table S5**). Hybrid indices ranged from 0.45 to 0.98 (**Figure 6A**). Only two groups (F_IT7 and W_IT10) were identified as hybrids, the four other groups (H_IT8, H_IT9, W_IT11 and W_AT12) had hybrid indices higher than 0.97 and were identified as *S. waldsteiniana*. Among the 1167 loci used for the analysis, 1147 (98%) were identified as outliers, representing 173 positive α, 318 negative α, 443 positive β and 715 negative β outliers (**Figure 6B**). Noticeably, 171 positive β outliers were also negative α outliers, but only 10 were also positive α outliers.

**Table 2:** Group statistics for *Salix foetida* and *Salix waldsteiniana* based on 128,200 loci.

Groups	PA	OH	GD	PI	FIS
F_FR1	0	0.19416	0.21208	0.22158	0.07844
F_FR2	0	0.20542	0.20241	0.21653	0.02902
F_FR3	0	0.20427	0.2016	0.22472	0.04492
F_CH4	33	0.16561	0.19096	0.20158	0.09378
F_CH5	2	0.1844	0.20225	0.21346	0.07857
F_AP6	7	0.17605	0.1739	0.18821	0.02918
F_IT7	650	0.22212	0.14138	0.14844	-0.11114
H_IT8	0	0.18182	0.21435	0.22521	0.11781
H_IT9	8	0.18397	0.21181	0.22244	0.10368
W_IT10	7	0.1934	0.213	0.22364	0.08112
W_IT11	78	0.19933	0.19084	0.20036	0.03075
W_AT12	0	0.20715	0.21542	0.22708	0.05472
W_SI13	7	0.1836	0.18883	0.20201	0.04539
W_AT14	0	0.2064	0.18019	0.20681	0.00316
W_SI15	0	0.17585	0.20383	0.21643	0.10719
W_MK16	0	0.16233	0.11328	0.15271	-0.01442

PA, number of private alleles; OH, Mean observed heterozygosity; GD, Gene diversity; PI, Nucleotide diversity; FIS, Inbreeding coefficient.

### 3.3 Morphometric analysis

*Salix alpina* and *S. breviserrata* were mostly differentiated from each other (**Supplementary Figure S9**), with highest differentiation by number of teeth (T). The PCA showed that hybrids had intermediate forms and fell within the phenotypic range of both parent species.

For *S. foetida* and *S. waldsteiniana*, the PCA showed substantial overlap of the parent species (**Supplementary Figure S10**). The number of teeth (T) was again the character that contributed most to the species differentiation. Hybrids showed intermediate phenotypes.

### 3.4 Ecological niche differentiation

**Figure 7:**
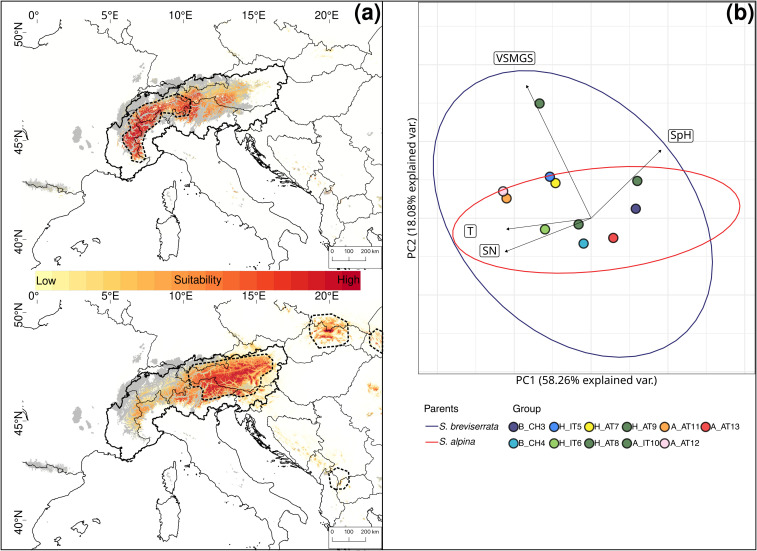
**(A)** Current habitat suitability based on six uncorrelated high-resolution climatic layers for *Salix breviserrata* (up) and *Salix alpina* (down). High suitability is indicated in dark red. The current distributions are indicated by the thick dashed outline. Areas covered by ice during the Last Glacial Maximum are indicated in grey. The Alps are delimited by the thick continuous outline. **(B)** Principal component analysis of environmental variables for *Salix breviserrata* and *Salix alpina*. Ellipses represent 95% confidence intervals for *Salix breviserrata* (blue) and *Salix alpina* (red) outside the contact zone. Colored dots represent the groups used for genetic analyses. Eigenvectors represent the 4 uncorrelated Landolt indicator values: Temperature (T), variability of soil moisture during the growing season (VSMGS), soil pH (SpH) and soil nutrient availability (SN). The European Commission, Eurostat (ESTAT), GISCO (2020). Countries, 2020 - Administrative Units - Dataset was used to draw the administrative boundaries **^©^** EuroGeographics.

The PCA for *S. alpina* and *S. breviserrata* indicated overlapping ecology between the two species (**Figure 7B**). *Salix breviserrata* had the most variable environmental niche and was found in a broader range of soil moisture (VSMGS), whereas *S. alpina* appeared on a broader range of soil nutrients. The niche overlap was 63% between the two species.

**Figure 8:**
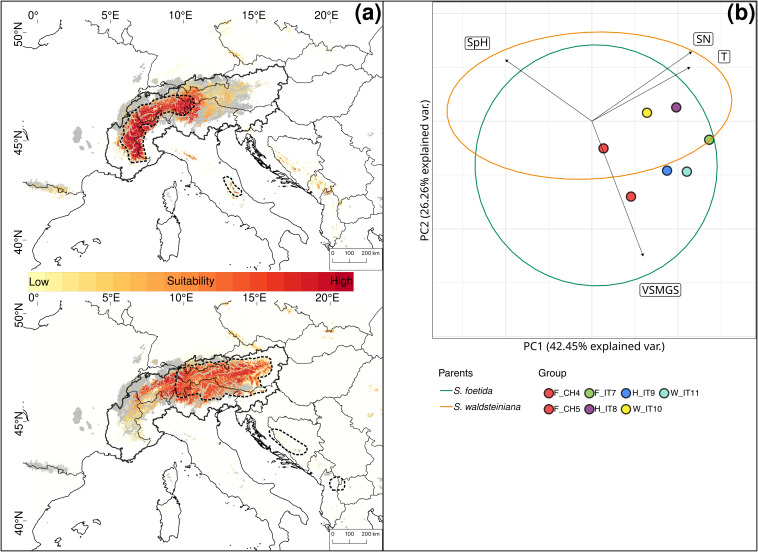
**(A)** Current habitat suitability based on six uncorrelated high-resolution climatic layers for *Salix foetida* (up) and *Salix waldsteiniana* (down). High suitability is indicated in dark red. The current distributions are indicated by the thick dashed outline. Areas covered by ice during the Last Glacial Maximum are indicated in grey. The Alps are delimited by the thick continuous outline. **(B)** Principal component analysis of environmental variables for *Salix foetida* and *Salix waldsteiniana*. Ellipses represent 95% confidence intervals for *Salix foetida* (green) and *Salix waldsteiniana* (orange) outside the contact zone. Colored dots represent the groups used for genetic analyses. Eigenvectors represent the 4 uncorrelated Landolt indicator values: Temperature (T), variability of soil moisture during the growing season (VSMGS), soil pH (SpH) and soil nutrient availability (SN). The European Commission, Eurostat (ESTAT), GISCO (2020). Countries, 2020 - Administrative Units - Dataset was used to draw the administrative boundaries **^©^** EuroGeographics.

The PCA for *S. foetida* and *S. waldsteiniana* showed overlapping ecological niches (**Figure 8B**). Soil moisture during the growing season (VSMGS) contributed most to a differentiation between the two species. The niche overlap was 41%.

### 3.5 Species distribution modelling

All single models were kept for the ensemble models. The ensemble model for each of the four species indicated high predictive accuracy with a TSS > 0.9. For *S. breviserrata*, projections in the LGM conditions identified potential glacial refugia at the foot of the Southwestern Alps, in Massif Central and in the Eastern Pyrenees (**Figure 3C**). For *S. alpina*, the projections found vast suboptimal areas in the periphery of the Eastern Alps and in the Carpathians (**Figure 3D**). *Salix foetida* had highly suitable conditions all along the Mediterranean coast, from the Pyrenees to the Alps, in the Southern Apennines (Italy) and in the Balkan Peninsula (**Figure 5C**). For *S. waldsteiniana*, suitable areas were mostly detected in the eastern part of the Alps, and in the Southern Carpathians (**Figure 5D**). According to current climate data, the projections of the highly suitable conditions were consistent with the current distribution for all four species (**Figures 7A**, **8A**). However, SDM predicted additional suitable habitats beyond the secondary contact zones.

## 4 Discussion

### 4.1 Biogeographical history of parent species

Alpine plant species in the European Alpine System survived the last glaciations in refugia, which provided a source for post-glacial recolonization (Hewitt, 1996). Notable peripheral refugial areas of the Alps include the Southwestern, Southern and Eastern Alps (Schönswetter et al., 2005). Additionally, extra-alpine origins have already been found for several alpine taxa (Moore and Kadereit, 2013; Surina et al., 2014; Kuzmanović et al., 2021). The present survey analyzed the genetic composition of two vicariant species pairs from their entire distribution areas to assess their biogeographical history. Analyses of gene flow and species distribution modeling suggest recolonization of the Alps from different potential refugial areas.

For *S. alpina*, our results suggest that the Tatra Mts. and Balkans harbored refugial populations, characterized by high numbers of private alleles (**Figures 1**, **3A**; **Table 1**). In accordance with other studies that covered the Carpathians and the Alps, we found some genetic differentiation between the two ranges (Mráz et al., 2007; Ronikier et al., 2008). However, biogeographical connections between the Eastern Alps and the Tatra Mts. are a common pattern in alpine plants (Schmitt, 2017). In *S. alpina*, gene flow from the Tatra Mts. towards the Alps highlights the connection between both regions (**Figure 3B**). The high number of Alpine-Carpathian endemics depicts the close relationship between both regions and supports our finding (Pawłowski, 1970). Colonization from the isolated North Macedonian group would have required long-distance dispersal (LDD) or the extinction of bridging populations in the northern Balkan Peninsula after recolonization, as suggested for *Wulfenia carinthiaca* (Surina et al., 2014). Indeed, to our knowledge, no other records exist for *S. alpina* in this region (Jalas and Suominen, 1988). A lack of phylogeographical differentiation within the Alps, as observed for *S. alpina*, was also found in *Oxytropis campestris* s.l. and *Hypochaeris uniflora* (Schönswetter et al., 2004; Patrik et al., 2007). Only one group of *S. alpina* in the Eastern Alps (A_AT11) has higher number of private alleles, lower gene diversity and lower nucleotide diversity compared to the other alpine groups (**Table 1**). This group is situated at the edge of the last glaciation ice sheet on an island-like mountain of carbonate rocks (Grebenzen), within an otherwise siliceous refugial area (Schönswetter et al., 2005) and could have survived the LGM in a marginal refugium. Its isolated position from other *S. alpina* populations allowed to keep its genetic distinctness. *Salix breviserrata* represents a monophyletic group deeply nested within the clade of *S. alpina* (**Figures 1**, **3B**). We suggest that the phylogenetic tree failed to separate the two species as sister clades due to broad introgression between the species pairs (see below). Hybridization has been shown to distort tree topologies (e.g (McDade, 1992)). A recent dated phylogeny suggests a split of *S. alpina* and *S. breviserrata* in the Pliocene (Marinček et al., 2024) and rejects the interpretation that *S. breviserrata* evolved out of *S. alpina* during the colonization of the Alps. Based on genetic results only, the potential glacial refugium for *S. breviserrata* remains unresolved. However, the projection of suitable conditions in the LGM identified a potential southwestern alpine peripheral refugium. The Maritime Alps are a known potential refugium (Taberlet et al., 1998), and evidences were found, e.g. for *Berardia subacaulis* and multiple *Gentiana* species (Christe et al., 2014; Guerrina et al., 2022). The samples from the Cantabrian Mts. appear on terminal branches of the phylogeny, suggesting a western range expansion from the Alps rather than a refugium, similar as in *Ranunculus parnassiifolius* (Cires and Prieto, 2012).

The refugial areas of *S. foetida* are unclear because the basal branch of the clade (**Figure 2**) is represented by a strongly differentiated group (F_IT7) in the Southern Alps, showing a high number of private alleles but low gene and nucleotide diversity. Excess of heterozygotes in this group support an interpretation as rare hybrid × hybrid genotypes, as observed in other willow hybrid zones (Gramlich et al., 2018). The low genetic diversity of this group (F_IT7) can be best explained by a drastic recent anthropogenic reduction of population size (covering just 15m^2^; see **Supplementary Table S3**) which has removed other, introgressed genotypes (see Marinček et al. (2023)). Another potential factor influencing genetic diversity within populations could be biased sex ratios in dioecious plants (Rosche et al., 2018), which would require further studies for our populations. For the other groups, the results could not identify any potential glacial refugium based on genetic statistics but the projection of suitable conditions in the LGM suggest a southwestern alpine peripheral refugium. The lack of distinct genetic structure and the partially low genetic diversity in both *S. breviserrata* and *S. foetida* confirm our hypothesis of rapid postglacial recolonization of the Alps. After the last glaciation maximum, both species rapidly colonized the newly available habitats, leading to a large area sharing similar genetic structure. Willow*s* are well-known pioneer species that rapidly colonize disturbed habitats and glacier forefields (Karrenberg et al., 2003; Gramlich et al., 2016). For *S. waldsteiniana*, results suggest a single refugial area. As for other species from the Eastern Alps, the refugium may have been located in the periphery or in the northern Dinaric Mts (Schönswetter et al., 2005; Durovic et al., 2017). Unexpectedly, the North Macedonian samples previously reported as *S. waldsteiniana* (Micevski, 1995) came out in genetic and morphometric analysis as hybrids with *S. foetida*. However, there is no other record of *S. foetida* in the Balkan Peninsula, but several ones for *S. waldsteiniana* (Jalas and Suominen, 1988). The genetic pattern might reflect a shared evolutionary history with *S. foetida* in the Central Apennines from Messinian or Pleistocene land bridge connections (Spaniel and Resetnik, 2022); similar distributions are also observed e.g. in *Saxifraga prenja* (Gerschwitz-Eidt et al., 2023). Our SDM suggests better habitat conditions for *S. foetida* than for *S. waldsteiniana* in the North Macedonia mountains, **Figure 8A**). Alternatively, LDD and hybridization events between *S. foetida* and other *S. waldsteiniana* populations (Jalas and Suominen, 1988) in the Balkan Peninsula cannot be excluded. Despite different potential refugia, postglacial recolonization of the sister species in the Alps happened mostly from eastern and southwestern marginal areas, with secondary contact hybridization in the Central Eastern and Southern Alps.

### 4.2 Secondary contact and hybrid zone

To understand the impact of hybridization on the biodiversity of the Alps, Parisod (2022) encouraged studies across suture zones where differentiated lineages come into sympatry. With the phylogeographic framework of the four species of interest (see above), we can follow the suggestion and investigate the two contact zones between the species pairs. Our study supplements other studies of hybrid zones in *Salix* (Hardig et al., 2000; Fogelqvist et al., 2015; Gramlich et al., 2016, 2018; Marinček et al., 2023) but explores for the first time hybrid zones over the whole distribution ranges. We reanalyzed the hybrid zone between *S. foetida* and *S. waldsteiniana* in a broader context than done previously (Marinček et al., 2023), and we newly characterized a homoploid hybrid zone of the species pair *S. alpina* and *S. breviserrata*. Our results suggest that secondary contact zones are species-specific, although the species pairs share identical, unspecialized pollination systems, efficient dispersal capabilities via wind-dispersed seeds, and similar biogeographical histories. In both cases we found introgressive hybridization which can be related to the low genetic divergence between the parental species pairs, as observed in other willows (Gramlich et al., 2018).

Both species pairs were represented by nonadmixed (parental) individuals dominating one marginal areas of the distribution, with hybrid individuals occurring in between. The morphometric data also confirm these findings. Other alpine plant species, as shown for *Androsace helvetica* and *Androsace pubescens*, likely originated in the northern Eastern Alps and Southwestern Alps, expanded their ranges toward the center of the Alps and hybridized after secondary contact (Schneeweiss et al., 2017). Typically, hybrid zones are described as “narrow regions” of hybridization (Barton and Hewitt, 1985; Harrison, 1993; Jiggins and Mallet, 2000). In this regard, we observed two distinct patterns. Specifically, *S. alpina* and *S. breviserrata* exhibited a broad hybrid zone, extending over more than 300 kilometers between the Zillertal Alps (Italy) and the Graz Mountains (Austria). Similar broad hybrid zones were found in *Fagus sylvatica* subsp. *orientalis*, which grows in the Caucasus and Asia Minor and in *Tephroseris helenitis* in the Eastern Alps (Pflugbeil et al., 2021; Hrivnák et al., 2024). The hybrid zone was larger than we expected based on morphology, because patterns of introgression into both parental species were found in individuals with phenotypes similar to pure parental individuals. Morphometric data confirmed that hybrids have a broad phenotypic variability and cover most of the parental variability (**Supplementary Figure S9**). Morphological variability is in willows strongly influenced by local habitat conditions (Neumann, 1981), and incongruences between phenotype and genetic admixture were already reported for *S. foetida* and *S. waldsteiniana* (Marinček et al., 2023). Overall, hybridization between *S. alpina* and *S. breviserrata* suggests the formation of hybrid swarms, as observed for other alpine species, e.g. in *Saxifraga* (Ebersbach et al., 2020). For *S. foetida* and *S. waldsteiniana*, the hybrid zone was narrower and situated between the Western and the Eastern Alps, on the line between Lake Garda and Innsbruck, known as the Brenner zone (Kerner, 1870; Thiel-Egenter et al., 2011). Our genetic data confirmed the presence of *S. waldsteiniana* and hybrids spread over 50 kilometers along the Brenner line, although it may extend to east Tyrol (Hörandl, 1992; not sampled here). Hybridization between *S. foetida* and *S. waldsteiniana* creates an asymmetric hybrid zone, where individuals belonging to one parental species co-occur with their hybrids.

The hybrid zones were further analyzed to characterize the hybrids and identify loci with exceptional patterns. For *S. alpina* and *S. breviserrata*, all individuals defined as hybrids in the analysis showed at least 16% admixture. The strong correlation between hybrid index and longitude confirmed the broad pattern of introgression rather than hybrid speciation or reinforcement, as found before in willows (Gramlich et al., 2018). All α outlier loci represented excess ancestry of *S. alpina.* This could be the result of a selective advantage towards homozygous genotypes from *S. alpina* in the secondary contact zone (Guo et al., 2023). Species distribution modelling in the present shows higher suitability in the secondary contact zone for *S. alpina* than *S. breviserrata*. Also, the ecology of the secondary contact zone is more similar to the ecological niche of *S. alpina*. All β outlier loci represented faster introgression than expected. No locus was identified as a positive β outlier that would potentially indicate reproductive isolation. These results confirmed the hypothesis of Hörandl that crossing barriers between the two species are low (Hörandl, 1992). The dominant pattern of introgression between the two closely related species and the high number of migration events found between groups suggest that gene flow might be sufficient to weaken species boundaries. In addition, there are no major differences in flowering times (Hörandl, 1992). As suggested by our ecological analysis, the habitat requirements for each species share similarities, with more than 60% of niche overlap. The availability of suitable habitats with both calcareous and base-rich siliceous substrates in the Eastern Alps may have enhanced the formation of a very broad and symmetric hybrid zone. Field observations and herbarium specimens within the hybrid zone (Hörandl, 1992) indicate normal catkin formation and seed set, i.e. no obvious postzygotic crossing barriers. If no postzygotic isolation can limit the interspecific hybridization, further hybridization and introgression might lead to the genetic homogenization of both species (Rieseberg and Carney, 1998).

In *S. foetida* and *S. waldsteiniana*, we observed an asymmetric hybrid zone. Asymmetry occurs when differences in gene flow are observed between the species or uneven barrier strength promotes the introgression in one direction more than in the other (Pickup et al., 2019). Both species have identical pollination and dispersal systems, and it is therefore very unlikely to observe asymmetric gene flow. Asymmetry could be due to differential introgression of adaptive loci versus loci involved in reproductive isolation (Suarez-Gonzalez et al., 2018). Asymmetric introgression could also arise from different abundance of parental species in the initial phases of the hybrid zone formation, as observed in a recently formed hybrid zone between *S. helvetica* and *S. purpurea* in the Western Alps (Gramlich et al., 2018). Our results rather suggest the presence of a stronger crossing barrier in *S. waldsteiniana*. Notably, 170 outlier loci indicating a slower introgression than expected also represented excess ancestry of *S. waldsteiniana* (against 10 representing excess of *S. foetida*). These loci are potentially indicative of reproductive isolation. In contrast, more loci indicating faster introgression and adaptive advantages also represented excess ancestry of *S. foetida.* Selection likely favored adaptation of *S. foetida* on habitats with more moist soils. Such habitats were in the Eastern Alps mostly available directly after the melting of the ice-shield on glacier forefields, where *S. foetida* is usually one of the first colonizers. In this time period, *S. foetida* was perhaps in the Eastern Alps more abundant than today and left introgressive signatures into *S. waldsteiniana*. After reforestation, *S. waldsteiniana* could have been the more efficient colonizer on drier sites like limestone screes, and in shrubberies of *Pinus mugo* and *Alnus alnobetula*, that are nowadays dominating the subalpine zone of the Northern and Southern ridges of the Eastern Alps but also are available more locally in the central parts (**Figure 8A**). More detailed studies on the recolonization process would be needed to support this hypothesis. The combination of data potentially explains the asymmetry of the hybrid zone and the presence of almost unadmixed *S. waldsteiniana* in the secondary contact zone (group W_IT11 detected by Structure, BGC and morphometrics as pure *S. waldsteiniana*).

### 4.3 Hypothetical impact of hybrid zones on the species’ recolonization of the Alps

The current projections for the four species revealed available suitable conditions beyond the hybrid zones. Only 30% of alpine plant species fill their ranges in the Alps almost completely (Dullinger et al., 2012b). Interestingly, all four alpine willow species included in this study (*S. herbacea, S. reticulata, S. retusa* and *S. serpillifolia*) filled their ranges between 88% and 100%. The range filling is positively correlated with an increase in dispersal capacity (Dullinger et al., 2012b). It is therefore not surprising that willows, known to be pioneer species that colonize open habitats showed almost complete range filling (Hörandl et al., 2012). Willows are specifically efficient as first colonizers of glacier forefields (Gramlich et al., 2016), and their adaptations to wind-dispersal of seeds also allows for long distance dispersal over mountain ridges. It is consequently unlikely that the topography of the European Alps impacts strongly the current recolonization of the four focal species and hinders complete range filling. Topographic barriers to recolonization were rather observed in genera that are not specifically adapted to wind-dispersal (e.g., in *Ranunculus kuepferi*, Kirchheimer et al. (2018)). Moreover, our ecological data suggest similar niches between sister species. Differentiation in soil preferences was often mentioned to characterize willow species (Martini and Paiero, 1988; Hörandl, 1992), and are in general a strong factor for explaining distributions of plants in the Eastern Alps (Alvarez et al., 2009). However, based on our ecological analysis, we believe that bedrock conditions alone cannot be regarded as a strong barrier to range filling. Accordingly, the hybrid zones do not fit a scenario of strong ecogeographical displacement of hybrids and parents, as suggested by Kadereit, but rather show clinal patterns of introgression (Kadereit, 2015). Other ecological factors like pollinators and phenology were not explored but are probably less important as the species of interest are both wind- and insect-pollinated, with unspecific flower morphology and main flowering time during June and July. The response to biotic and abiotic stress factors with secondary compounds, as a potential factor, was found to be very similar for the species pair *S. foetida* and *S. waldsteiniana* (Volf et al., 2023).

Eventually, the prevalence of admixed ancestry in the secondary contact zone could be interpreted as a higher fitness of hybrid populations in comparison with parent species (Arnold and Martin, 2010). Genetic patterns matching later-generation hybrids in both hybrid zones are expected when F1 hybrids are fertile and indicate no fitness reduction in hybrids (Gramlich et al., 2016, 2018). According to our field observations, hybrids of our species pairs do form fertile catkins and seeds, but Hörandl (1992) reported for intermediate forms sometimes aborted catkins in herbarium collections. However, these observations are for both species’ pairs insufficient to draw conclusions about hybrid fitness.

In conclusion, we suggest that the biogeographical history and ecological bedrock conditions shaped the distribution of the willow species pairs, as found in other alpine species (Alvarez et al., 2009). In the secondary contact zone, hybrid zones could have influenced the recolonization process by introgression. Further studies on reproductive interference would be needed to test this hypothesis, whereby changes in hybridization dynamics over time have to be considered (Kyogoku, 2020). Our present data suggest that the incomplete range filling could have been influenced by the high genetic similarity between sister species and the hybridization that ensues from it. We propose that parent species have not yet recolonized the Alps beyond the secondary contact zone because the genetically similar sister species has already occupied suitable habitats. Other ecological factors hindering range filling, such as continued reforestation, the up-moving of alpine species due to recent global warming (Dullinger et al., 2012a), and microclimatic conditions, have to be studied. However, our work provides a new perspective on the importance of hybrid zones as an evolutionary and biogeographical process and highlights the significance of considering and studying species across their entire distribution using multiple methods.

## Data Availability

The datasets presented in this study can be found in online repositories. The names of the repository/repositories and accession number(s) can be found in https://www.ncbi.nlm.nih.gov, PRJNA873074.

## Supplementary material

Supplementary Figure 1 Map of the study area with different mountain systems of Europe.

Supplementary Figure 2 Bioinformatic workflow to extract and filter loci for downstream analyses.

Supplementary Figure 3 Evanno analysis plots for *Salix alpina* and *Salix breviserrata*.

Supplementary Figure 4 Evanno analysis plots for *Salix foetida* and *Salix waldsteiniana*.

Supplementary Figure 5 Result of the principal component analysis for *Salix breviserrata* and *Salix alpina*.

Supplementary Figure 6 Result of the principal component analysis for *Salix foetida* and *Salix waldsteiniana*.

Supplementary Figure 7 Likelihood, variance explained, and Delta m values derived from TreeMix analysis for *Salix alpina* and *Salix breviserrata*.

Supplementary Figure 8 Likelihood, variance explained, and Delta m values derived from TreeMix analysis for *Salix foetida* and *Salix waldsteiniana*.

Supplementary Figure 9 Principal component analysis of morphological characters for *Salix alpina* and *Salix breviserrata*.

Supplementary Figure 10 Principal component analysis of morphological characters for *Salix foetida* and *Salix waldsteiniana*.

Supplementary Table 1 Location and sampling details of the 352 accessions included in the article.

Supplementary Table 2 Leaves measurements used for morphometric analysis.

Supplementary Table 3 Vegetation relevés from 19 sampling sites.

Supplementary Table 4 Pairwise Fst values for the groups of *Salix alpina* and *Salix breviserrata*.

Supplementary Table 5 Pairwise Fst values for the groups of *Salix foetida* and *Salix waldsteiniana.*
