# Supplementary material for: Hybrid zones in the European Alps impact the phylogeography of alpine vicariant willow species (Salix L.)
Source: Front Plant Sci. 2025 Mar 20;16:1507275. doi: 10.3389/fpls.2025.1507275 (PMC11966204; doi:10.3389/fpls.2025.1507275)
Supplement: Supplementary Figure 1 — Map of the study area with different mountain systems of Europe. [file DataSheet1.pdf]

Supporting Material

Hybrid zones in the European Alps impact the phylogeography of alpine vicariant willow species (Salix L.)

Loïc Pittet, Pia Marinček, Piotr Kosiński, Natascha D. Wagner and Elvira Hörandl

The following Supporting Information is available for this article:

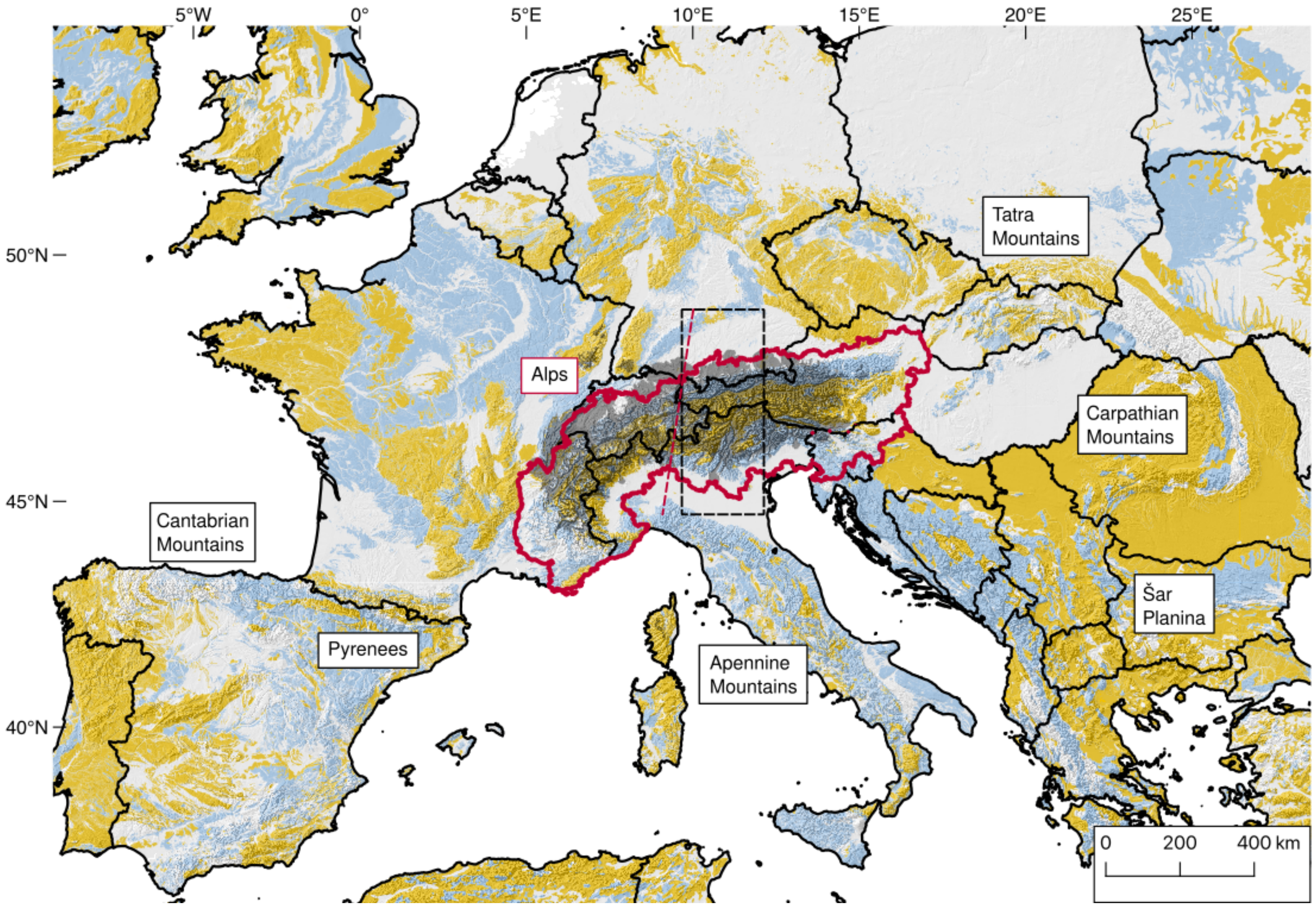

Figure S1: Map of the study area with different mountain systems of Europe. The European Alps are delimited by the continuous red line. The Western Alps and Eastern Alps are separated by the dashed line in red, according to Schönswetter et al. (2005). The secondary contact zone is represented by the dashed rectangle in black. Yellow, blue and grey background colors represent siliceous, calcareous and mixed substrate, respectively. The dark color inside the European Alps represents the ice shield extent during the Last Glacial Maximum.

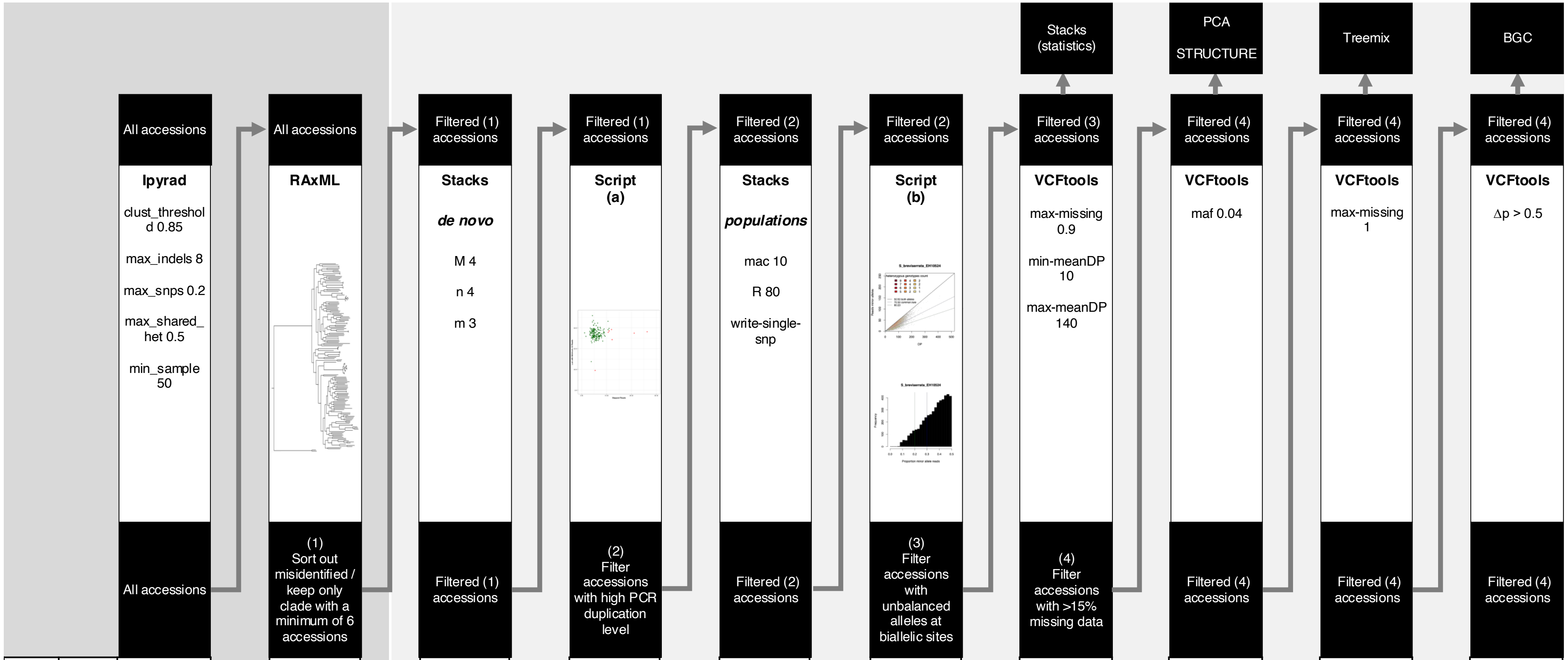

S. alpina S. breviserrata	N	162	156	156	152	152	152	152	152	152	152
	SNPs	598,650	598,650	1,564,100	1,564,100	48,071	48,071	40,126	35,858	6,455	526
S. foetida S. waldsteinana	N	161	147	147	141	141	140	140	140	140	140
	SNPs	575,194	575,194	1,176,008	1,176,008	48,565	48,565	39,365	36,533	5,384	1,197

Figure S2: Bioinformatic workflow to extract and filter loci for downstream analyses. In the dark grey box, IPYRAD was used to generate a preliminary dataset that was used to create a phylogenetic tree that was used to sort out misidentifications and create a population map that will be used with STACKS. In the light grey box, STACKS was used to generate the final dataset that will be used for downstream analysis. The

denovo_map.pl and populations program were used. Tests were made to remove accessions with high proportion of PCR duplicates (script (a); https://github.com/joanam/scripts/blob/master/createRADmappingReport.sh) and to remove contaminated accessions (script (b); https://github.com/speciationgenomics/scripts/blob/master/checkHetsIndvVCF.sh). Finally, the data were further filtered to match the requirements according to each downstream analysis. The numbers of accessions and SNPs retained at each step and for each species pair are displayed in the table below the workflow.

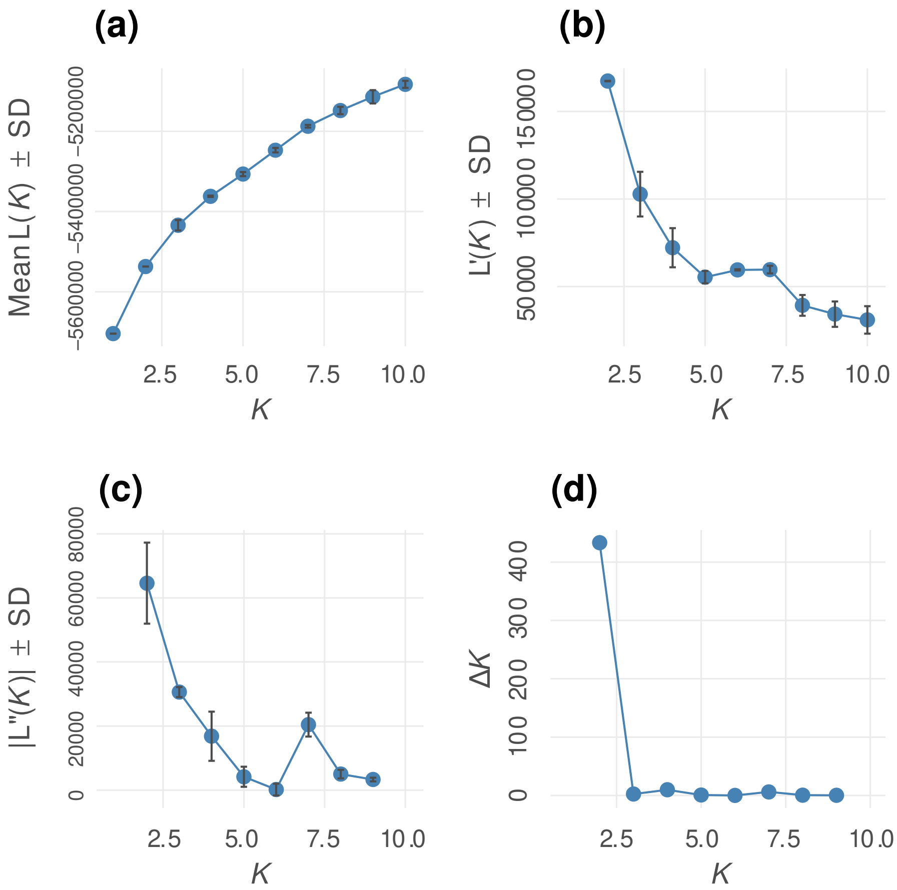

Figure S3: Evanno analysis plots for *Salix alpina* and *Salix breviserrata*. **(A)** Estimated log probability of data over increasing values of K. **(B)** First derivate of the estimate log probability. **(C)** Second derivate of the estimate log probability. **(D)** Delta K over values of K.

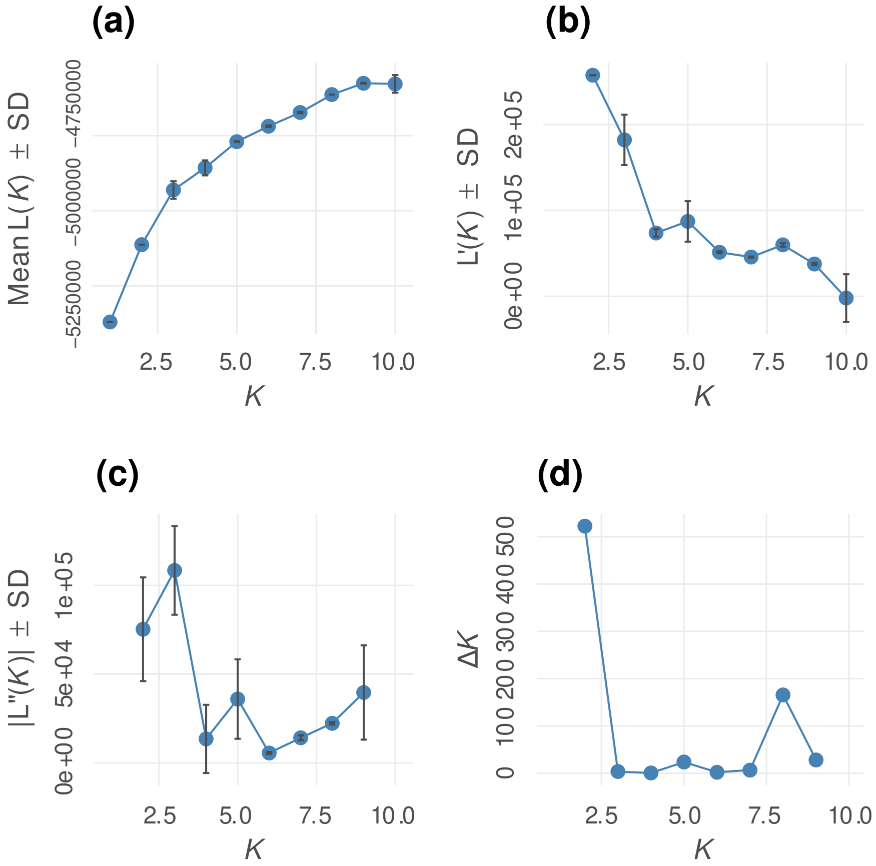

Figure S4: Evanno analysis plots for *Salix foetida* and *Salix waldsteiniana*. **(A)** Estimated log probability of data over increasing values of K. **(B)** First derivate of the estimate log probability. **(C)** Second derivate of the estimate log probability. **(D)** Delta K over values of K.

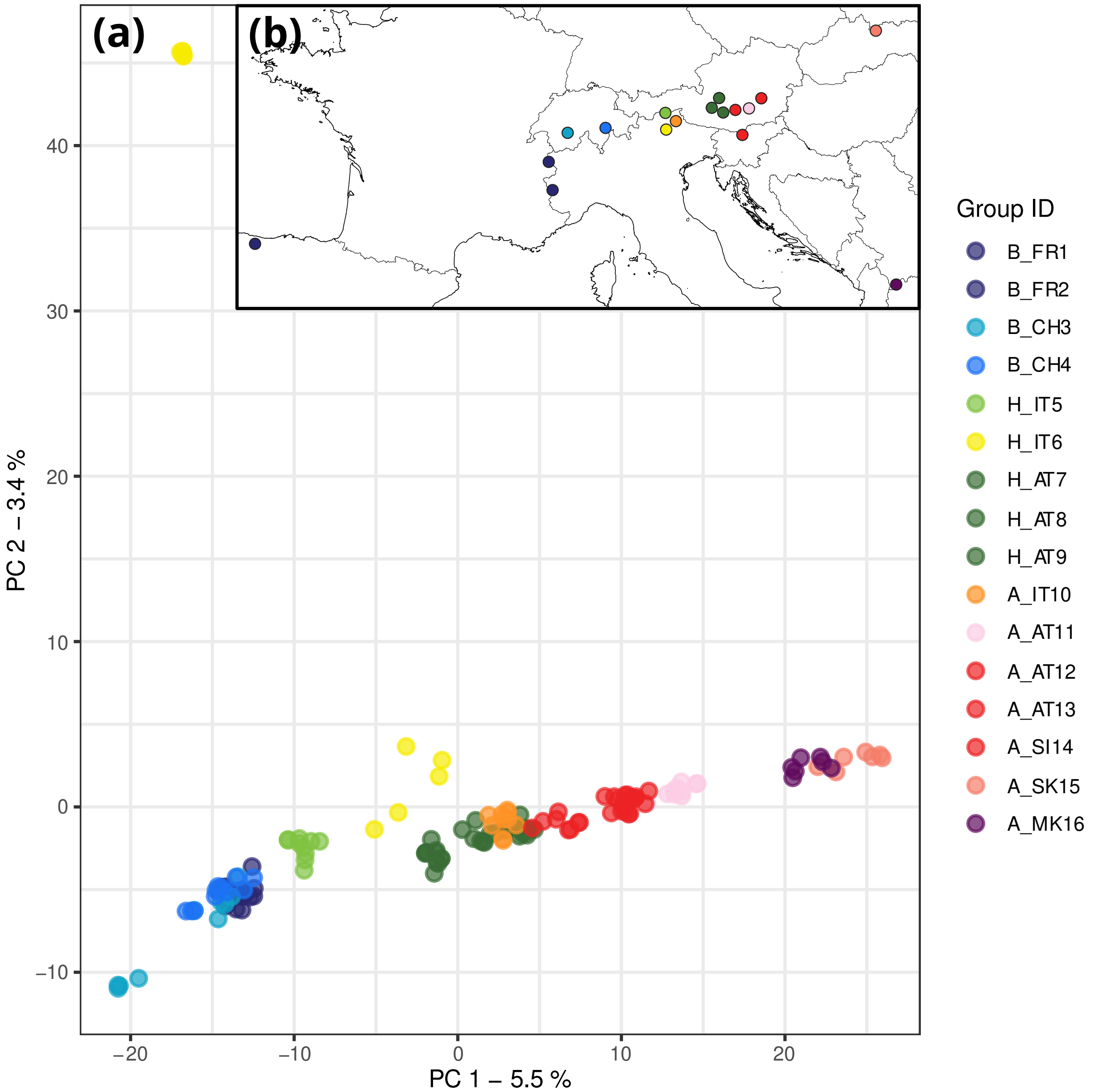

Figure S5: (A) Scatterplot showing the result of the principal component analysis based on 35,858 unlinked loci for 152 individuals (16 groups) for *Salix breviserrata* and *Salix alpina*. **(B)** Map with the location of the 16 groups.

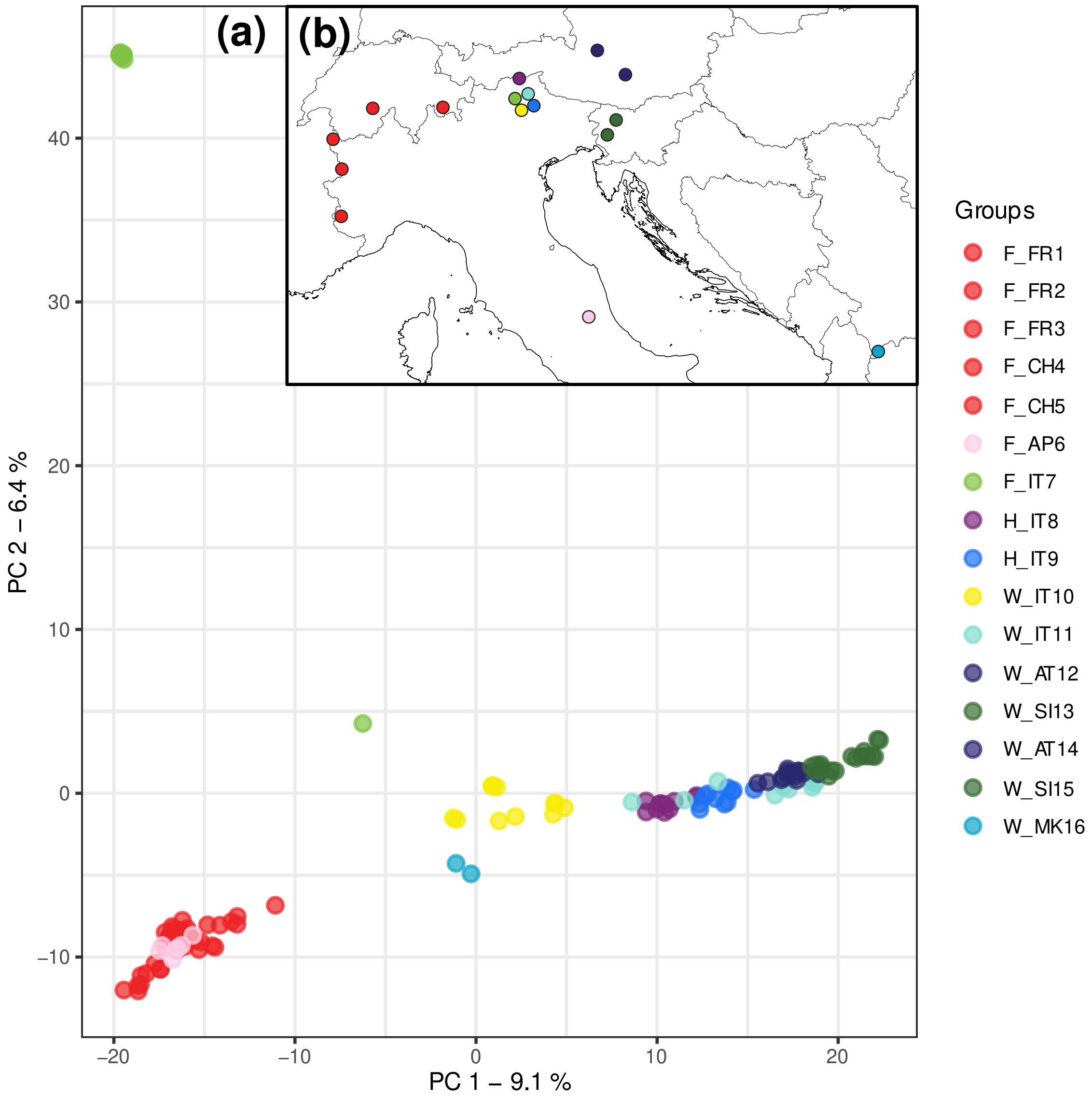

Figure S6: (A) Scatterplot showing the result of the principal component analysis based on 36,533 unlinked loci for 140 individuals (16 groups) for *Salix foetida* and *Salix waldsteiniana*. **(B)** Map with the location of the 16 groups.

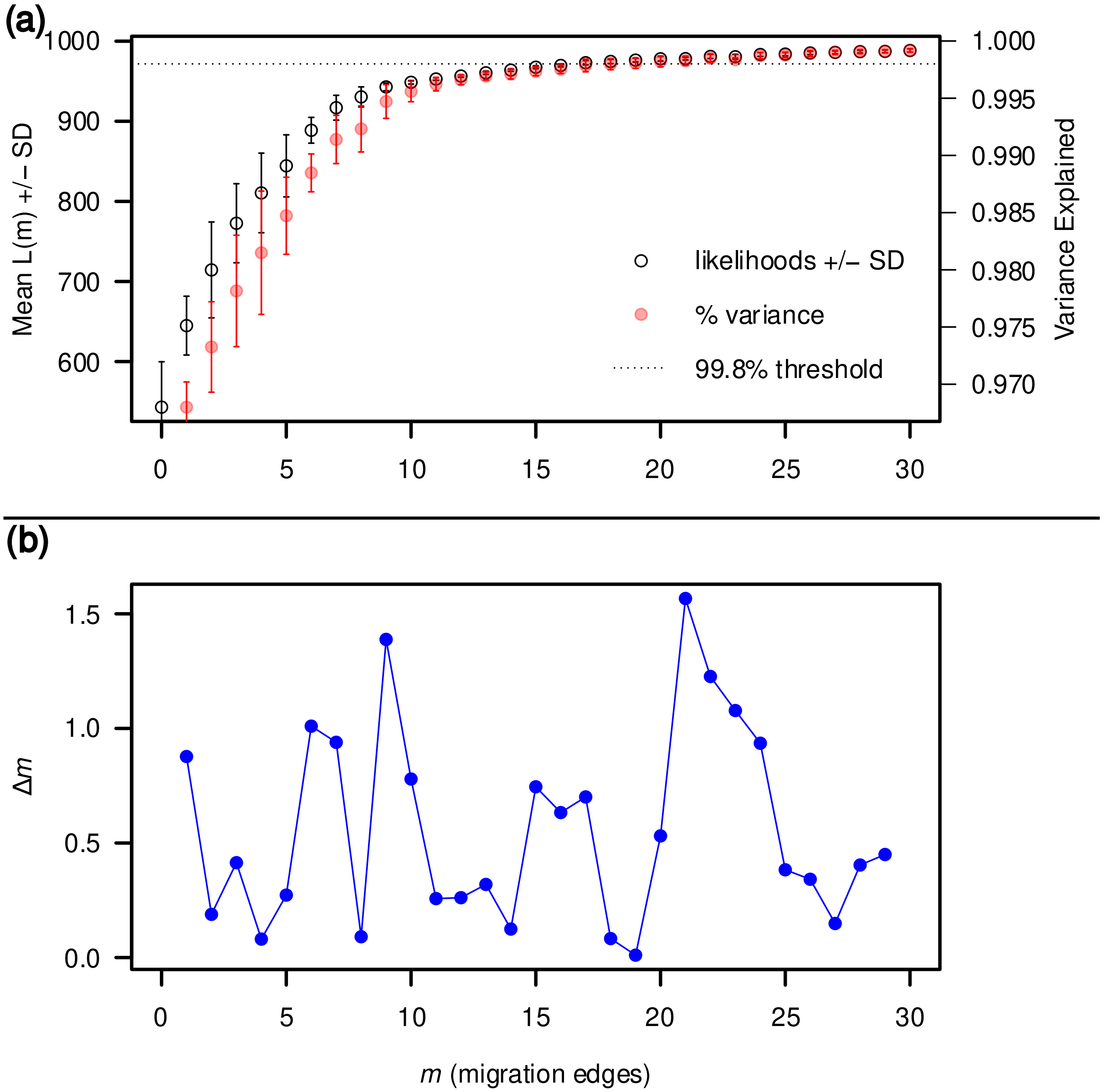

Figure S7: (A) Likelihood (white dot: mean and standard deviation) and variance explained (red dot: percentage). **(B)** Delta m values derived from TREEMIX analysis for *Salix alpina* and *Salix breviserrata*.

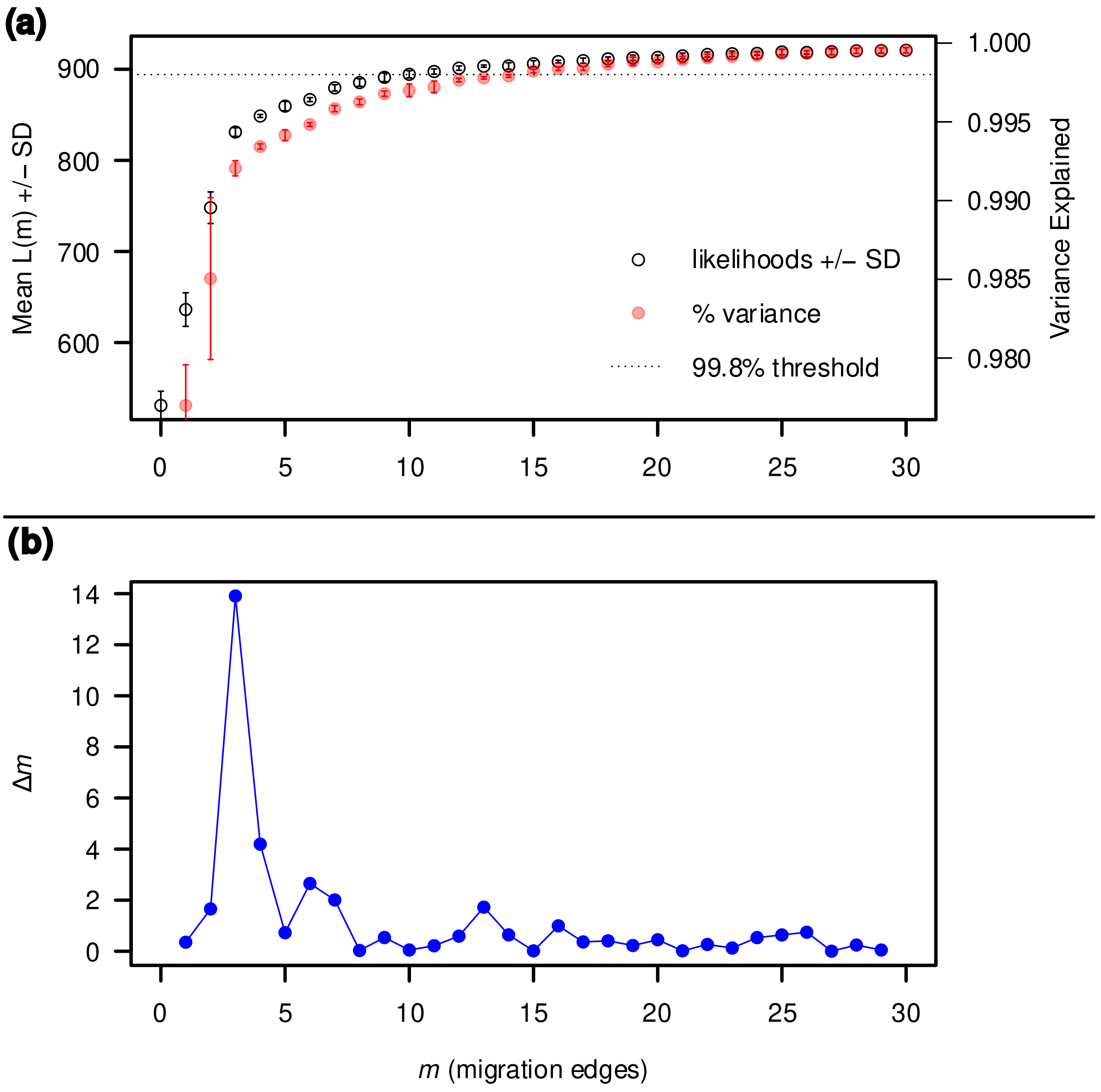

Figure S8: (A) Likelihood (white dot: mean and standard deviation) and variance explained (red dot: percentage). **(B)** Delta m values derived from TREEMIX analysis for *Salix foetida* and *Salix waldsteiniana*.

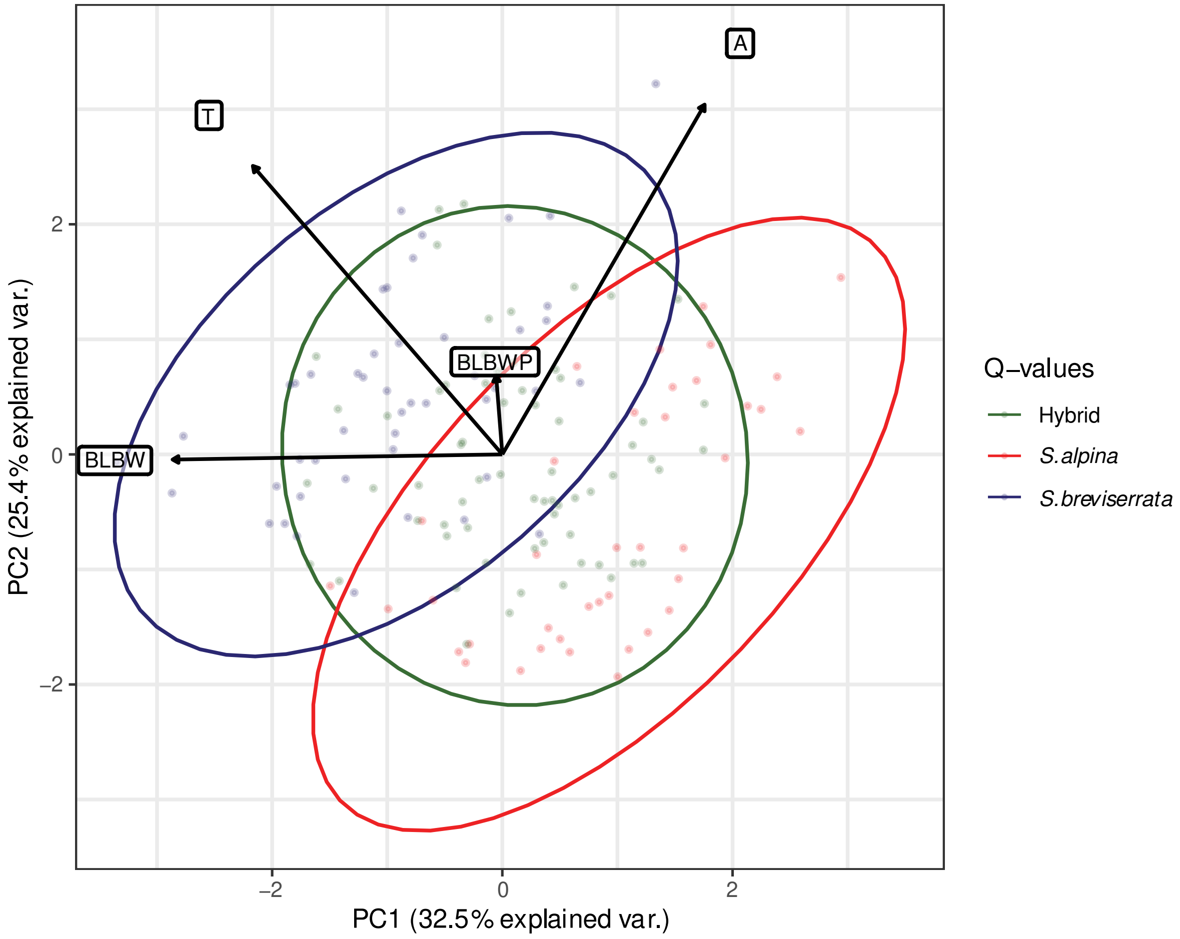

Figure S9: Principal component analysis of morphological characters for *Salix alpina* and *Salix breviserrata*. Ellipses represent 95% confidence intervals for *Salix alpina* (red), *Salix breviserrata* (blue) and individuals showing more than 10% admixture in the STRUCTURE analysis (green). Eigenvectors represent the 4 uncorrelated metrics: Area (A), total number of teeth (T), leaf length to width ratio (BLBW) and ratio of leaf total length to length of the widest position (BLBWP).

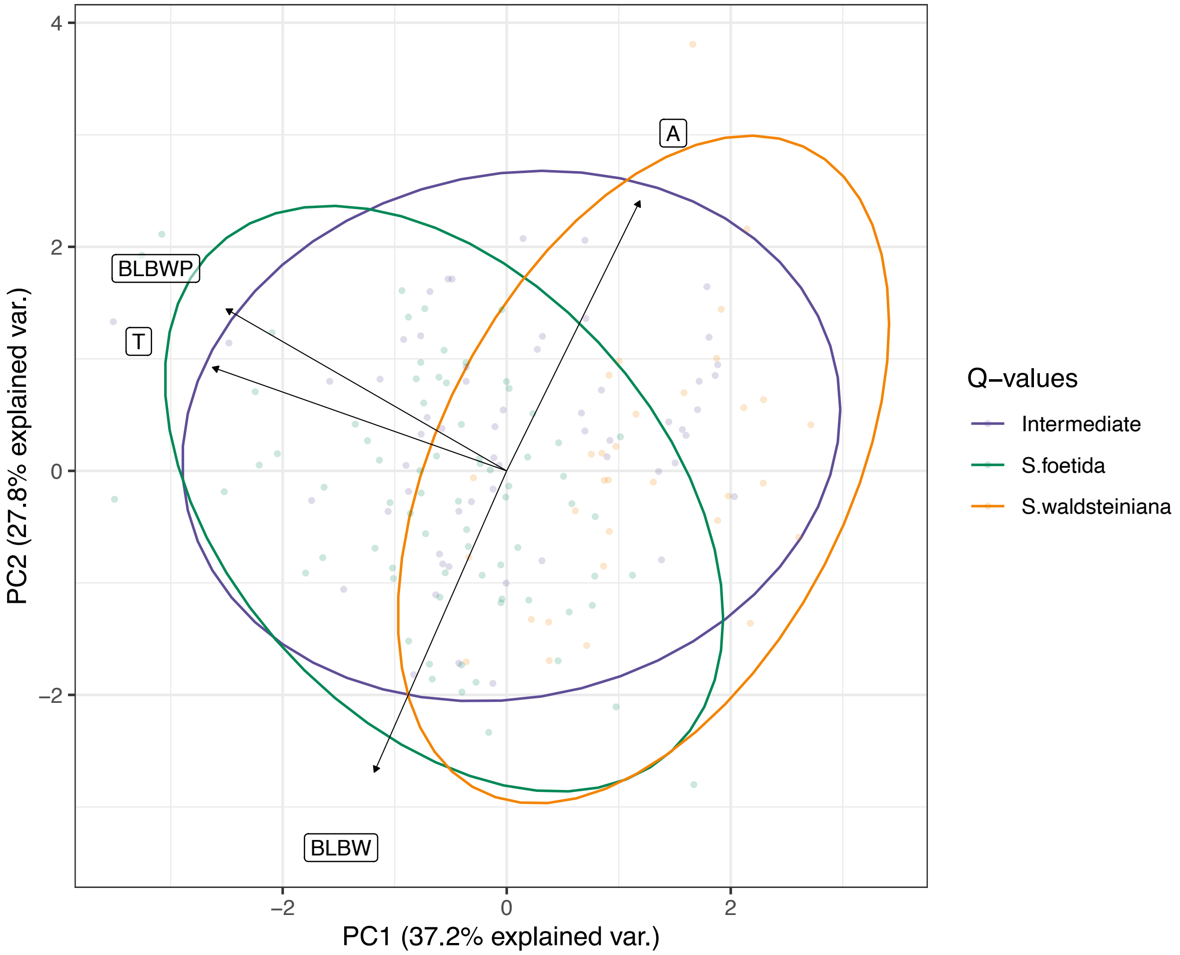

Figure S10: Principal component analysis of morphological characters for *Salix f*oetida and *Salix waldsteiniana*. Ellipses represent 95% confidence intervals for *Salix foetida* (green), *Salix waldsteiniana* (orange) and individuals showing more than 10% admixture in the STRUCTURE analysis (blue). Eigenvectors represent the 4 uncorrelated metrics: Area (A), total number of teeth (T), leaf length to width ratio (BLBW) and ratio of leaf length to length widest position (BLBWP).

Table S1 (Excel sheet): Location and sampling details of the 352 accessions included in the article. Sample ID, phenotype, location ID, group ID, locality by country with ISO code 3166-2, state, region, altitude (in meter above sea level, m.a.s.l.), latitude (N, decimal), longitude (E, decimal), collection date, collector, herbarium voucher specimens, and summary of accessions inclusion across different analyses.

Table S2 (Excel sheet): Leaves measurements used for morphometric analysis. We measured 2,791 leaves representing 317 accessions. Sample ID, Group ID, Genetic classification (Based on STRUCTURE Q-values; A,B,F,W for *Salix alpina, Salix breviserrata, Salix foetida, Salix waldsteiniana*, respectively. H for hybrids), Phenotype (based on field observation; A,B,F,W for *Salix alpina, Salix breviserrata, Salix foetida, Salix waldsteiniana,* respectively. I for intermediate form), latitude (N, decimal), longitude (E, decimal), altitude (in meter above sea level, m.a.s.l.), surface area (A), perimeter (P), number of teeth (T), length (BL), width (BW), position of maximal width (BWP), position of maximal width in percentage of BL (LBW), ratio surface to perimeter (A/P), ratio length to width (BL/BW), ration length to width position (BL/BWP), ratio number of teeth to perimeter (T/P).

Table S3 (Excel sheet): Vegetation relevés from 19 sampling sites. All relevés were done by E.H., following the Braun-Blanquet method. Moss and lichen species were not determined. Group ID, relevé date, locality, latitude (N, decimal), longitude (E, decimal), altitude (in meter above sea level, m.a.s.l.), size of the relevé (m), exposition, measured pH, potential pH, slope (in percentage), vegetation type, coverage (in percentage), list of co-occurring species and corresponding presence indices.

	B_FR2	B_CH3	B_CH4	H_IT5	H_IT6	H_AT7	H_AT8	H_AT9	A_IT10	A_AT11	A_AT12	A_AT13	A_SI14	A_SK15	A_MK16
B_FR1	0.046	0.057	0.052	0.048	0.073	0.069	0.064	0.059	0.060	0.090	0.071	0.075	0.067	0.124	0.120
B_FR2		0.062	0.055	0.051	0.075	0.072	0.066	0.061	0.062	0.094	0.074	0.078	0.069	0.129	0.125
B_CH3			0.065	0.061	0.084	0.082	0.076	0.071	0.072	0.103	0.084	0.088	0.080	0.138	0.134
B_CH4				0.052	0.076	0.074	0.069	0.064	0.064	0.094	0.075	0.080	0.071	0.126	0.123
H_IT5					0.066	0.065	0.058	0.054	0.053	0.083	0.064	0.067	0.060	0.113	0.110
H_IT6						0.083	0.075	0.071	0.069	0.097	0.080	0.082	0.074	0.127	0.124
H_AT7							0.066	0.061	0.065	0.090	0.070	0.074	0.065	0.119	0.117
H_AT8								0.052	0.053	0.075	0.057	0.060	0.051	0.102	0.100
H_AT9									0.051	0.073	0.054	0.057	0.049	0.099	0.096
A_IT10										0.074	0.056	0.057	0.047	0.099	0.098
A_AT11											0.078	0.076	0.067	0.120	0.120
A_AT12												0.062	0.052	0.109	0.107
A_AT13													0.051	0.104	0.104
A_SI14														0.091	0.090
A_SK15															0.147

Table S4: Pairwise Fst values for the groups of *Salix alpina* and *Salix breviserrata*.

	F_FR2	F_FR3	F_CH4	F_CH5	F_AP6	F_IT7	H_IT8	H_IT9	W_IT10	W_IT11	W_AT12	W_SI13	W_AT14	W_SI15	W_MK16
F_FR1	0.037	0.038	0.056	0.048	0.067	0.131	0.069	0.074	0.064	0.095	0.073	0.100	0.090	0.086	0.082
F_FR2		0.055	0.069	0.061	0.086	0.154	0.078	0.084	0.074	0.107	0.083	0.116	0.114	0.098	0.116
F_FR3			0.069	0.062	0.095	0.160	0.075	0.081	0.072	0.105	0.079	0.117	0.124	0.096	0.140
F_CH4				0.076	0.101	0.167	0.091	0.096	0.087	0.119	0.095	0.127	0.125	0.110	0.126
F_CH5					0.089	0.153	0.079	0.085	0.076	0.107	0.083	0.113	0.109	0.097	0.109
F_AP6						0.190	0.100	0.106	0.097	0.131	0.105	0.145	0.154	0.123	0.172
F_IT7							0.146	0.151	0.142	0.176	0.145	0.186	0.197	0.164	0.231
H_IT8								0.057	0.064	0.081	0.049	0.073	0.069	0.058	0.093
H_IT9									0.067	0.077	0.050	0.074	0.070	0.059	0.098
W_IT10										0.090	0.061	0.087	0.081	0.072	0.098
W_IT11											0.074	0.101	0.099	0.084	0.125
W_AT12												0.059	0.057	0.044	0.091
W_SI13													0.092	0.068	0.145
W_AT14														0.070	0.192
W_SI15															0.114

Table S5: Pairwise Fst values for the groups of *Salix foetida* and *Salix waldsteiniana*.

References:

Schönswetter P., Stehlik I., Holderegger R., Tribsch A. 2005**.** Molecular evidence for glacial refugia of mountain plants in the European Alps. *Mol Ecol* 14: 3547-3555.
